# Effects of cannabinoids on ligand-gated ion channels

**DOI:** 10.3389/fphys.2022.1041833

**Published:** 2022-10-19

**Authors:** Murat Oz, Keun-Hang Susan Yang, Mohamed Omer Mahgoub

**Affiliations:** ^1^ Department of Pharmacology and Therapeutics, Faculty of Pharmacy, Kuwait University, Kuwait City, Kuwait; ^2^ Department of Biological Sciences, Schmid College of Science and Technology, Chapman University, One University Drive, Orange, CA, United States; ^3^ Department of Health and Medical Sciences, Khawarizmi International College, Abu Dhabi, UAE

**Keywords:** cannabinoids, endocannabinoids, synthetic cannabinoids, ligand-gated ion channels, ion channels

## Abstract

Phytocannabinoids such as Δ^9^-tetrahydrocannabinol and cannabidiol, endocannabinoids such as N-arachidonoylethanolamine (anandamide) and 2-arachidonoylglycerol, and synthetic cannabinoids such as CP47,497 and JWH-018 constitute major groups of structurally diverse cannabinoids. Along with these cannabinoids, CB1 and CB2 cannabinoid receptors and enzymes involved in synthesis and degradation of endocannabinoids comprise the major components of the cannabinoid system. Although, cannabinoid receptors are known to be involved in anti-convulsant, anti-nociceptive, anti-psychotic, anti-emetic, and anti-oxidant effects of cannabinoids, in recent years, an increasing number of studies suggest that, at pharmacologically relevant concentrations, these compounds interact with several molecular targets including G-protein coupled receptors, ion channels, and enzymes in a cannabinoid-receptor independent manner. In this report, the direct actions of endo-, phyto-, and synthetic cannabinoids on the functional properties of ligand-gated ion channels and the plausible mechanisms mediating these effects were reviewed and discussed.

## Introduction

Cannabis sativa plant (marijuana) consists of more than 500 chemical compounds, and about 120 of them are structurally related phytocannabinoids which are terpeno-phenolic compounds chemically related to the terpenes with their ring structure derived from a geranyl pyrophosphate (ElSohly et al., 2017). Among these phytocannabinoids, Δ^9^-tetrahydrocannabinol (THC) and cannabidiol (CBD) are major psychoactive and non-psychoactive components of marijuana, respectively (Izzo et al., 2009).

Following structural characterization of series of phytocannabinoids in 1960s, CB1 and CB2 cannabinoid (CB) receptors, and endocannabinoids anandamide (N-arachidonoylethanolamine; AEA) and 2-arachidonoylglycerol (2-AG) were identified and shown to activate CB1 and CB2 with high affinity and efficacy (Devane et al., 1992; Mechoulam et al., 1995; Cristino et al., 2020). Subsequently, enzymes involved in synthesis and inactivation of endocannabinoids were identified. Synthesis of AEA and other N-acylethanolamines is catalyzed by N-acylphosphatidylethanolamine (NAPE)-specific phospholipase D-like hydrolase (NAPE-PLD), and biosynthesis of 2-AG and other monoacylglycerols are catalyzed by Diacylglycerol lipase α (DAGLα) and DAGLβ. On the other hand, while anandamide and other N-acylethanolamines are hydrolyzed by fatty acid amide hydrolase (FAAH), the hydrolysis of 2-AG and other monoacylglcerols catalyzed by monoacylglycerol lipase (MAGL) (Fowler et al., 2017; Cristino et al., 2020). This system of endogenous ligands, receptors and metabolic enzymes became known as the endocannabinoid system. In addition to phyto- and endocannabinoids, intensive structure-activity studies to synthetize novel high-affinity ligands for cannabinoid receptors have resulted in emergence of several compounds such as WIN55,212-2, HU210, and CP55,940 with significantly diverse chemical structures that constitute third group of cannabinoids collectively coined synthetic cannabinoids (Huffman and Padgett, 2005; Wiley et al., 2014; Le Boisselier et al., 2017; Howlett et al., 2021).

In recent years, lack of psychoactive effects of some phytocannabinoids such as CBD, increased access to cannabis products due to decriminalization of medical use of cannabis in several western countries, and new approvals for clinical use of cannabinoid-based compounds have revitalized the interest in pharmacological effects of phytocannabinoids, specifically the CBD. In clinical practice, Nabilone (Cesamet), a synthetic cannabinoid, and Dronabinol (Marinol), synthetic (-) enantiomer of THC, have been used for treatment of anorexia and chemotherapy-induced nausea and vomiting in the United States, Canada, and United Kingdom (Wright and Guy, 2014; Pagano et al., 2022). Nabiximols (Sativex), an oral spray containing CBD and THC in a 1: 1 ratio, has been approved in several countries including United Kingdom, European Union, and Canada for the treatment of multiple sclerosis-associated spasticity (Ortiz et al., 2022; Pagano et al., 2022). CBD has been recently approved in the United States and the European Union as an add-on antiepileptic drug (Epidiolex) for the treatment of patients affected by refractory epilepsy such as Dravet syndrome and Lennox–Gastaut syndrome (Ortiz et al., 2022; Pagano et al., 2022) and caused resurgence of interest in the pharmacology of cannabinoids in general and phytocannabinoids in particular.

Since CBD does not activate CB1 and CB2 receptors, some of the attention in cannabinoid research has recently been focused on CB1 and CB2-independent cellular and molecular targets for CBD and other phytocannabinoids such as cannabigerol and cannabidivarin (Ghovanloo et al., 2022; Isaev et al., 2022; Mirlohi et al., 2022). In fact, in earlier studies, cannabinoid-receptor independent effects have been described for both endogenous and synthetic cannabinoids as well (Johnson et al., 1993; Fan, 1995; Oz et al., 2000). AEA stimulates GTPγS binding in brain membranes isolated from mice lacking CB1 receptors, and this effect is not altered by CB1 and CB2 antagonists (Di Marzo et al., 2000). THC and AEA-induced analgesic effects in hot-plate (for AEA) and tail flick tests (for THC) remain intact in CB1 knock-out (Zimmer et al., 1999; Di Marzo et al., 2000) or both CB1 and CB2 knock-out mice (Rácz et al., 2008). Similarly, AEA still induces catalepsy and analgesia and decreases spontaneous activity in CB1-deficient mice (Di Marzo et al., 1994; Baskfield et al., 2004) and exert CB1 antagonist-resistant behavioral effects in the open-field test (Järbe et al., 2003a; Järbe et al., 2003b).

In neuronal systems, fast signal transmission between pre and post synaptic structures is carried out by the conformational changes induced by the signaling molecules, neurotransmitters in integral membrane proteins coined ligand-gated ion channels (LGICs). The LGICs are divided into three super families: the Cys-loop superfamily, the glutamate receptors [NMDA (*N*-methyl-D-aspartate), AMPA (*α*-amino-3-hydroxy-5-methyl-4-isoxazolepropionic acid) and kainate], and the ATP-gated channels (Alexander et al., 2017). The Cys-loop superfamily comprises both cationic receptors such as nicotinic acetylcholine (nACh) and 5-HT_3_ receptors and anionic receptors such as GABA (*γ* -aminobutyric acid) type A (GABA_A_), and glycine receptors. Typical LGIC is composed of multiple subunits in homo or hetero pentameric structure with a pore forming α subunit that allows the regulated flow of ions across the plasma membrane in response to binding of endogenous or exogenous molecules. Structurally, an α-subunit of LGIC contains a large extracellular N-terminus, four transmembrane (TM) domains, a short extracellular C-terminus, and a large cytoplasmic domain between TM3 and TM4 (Plested, 2016). While this review focuses on the direct cannabinoid receptor-independent effects of cannabinoids on the LGICs, the effects of these compounds on other ion channels, various G-protein coupled receptors, enzymes, and neurotransmitter transporters have been reviewed in earlier reports (Oz, 2006; Pertwee et al., 2010; Soderstrom et al., 2017; Cifelli et al., 2020; Senn et al., 2020; Vitale et al., 2021).

### Effects of cannabinoids on nicotinic acetylcholine receptors

Nicotinic acetylcholine receptors (nAChRs) are cationic channels belonging to the Cys-loop superfamily of ligand-gated ion channels and their opening is controlled by the endogenous neurotransmitter ACh or exogenous ligands such as nicotine (Albuquerque et al., 2009; Dineley et al., 2015). They are homo or hetero-pentameric complexes comprised of α1-10, β1-6, δ, and γ subunit combinations and mediate fast cholinergic neurotransmission in both central and peripheral nervous systems and are expressed in non-neuronal cells as well (Albuquerque et al., 2009; Zoli et al., 2015). The homomeric α7-nAChR subtype plays an important role in synaptic plasticity and various disease pathologies including pain, neuroinflammation, and neurodegenerative diseases such as Alzheimer and Parkinson diseases (Oz et al., 2013; Lorke et al., 2016; Oz et al., 2016; Borroni and Barrantes, 2021; Mizrachi et al., 2021; Papke and Horenstein, 2021).

In *vivo* studies, WIN55,212-2, non-selective cannabinoid receptor agonist, has been shown to impair memory-related effects of nicotine in male rats (Robinson et al., 2010) and mice (Biala and Kruk, 2008). In *Xenopu*s oocytes expressing α7-nACh receptors, AEA and 2-AG noncompetitively inhibited α7-nACh receptor-mediated currents with IC_50_ values of 229 and 168 nM, respectively (Oz et al., 2003; Oz et al., 2004b). In line with these findings, radioligand binding studies indicated that AEA, at concentration range of 30-300 μM, does not alter specific binding of [^3^H]nicotine in human frontal cortex (Lagalwar et al., 1999) and rat thalamic membranes (Butt et al., 2008), further suggesting a noncompetitive nature of AEA effect on nACh receptors. Inhibition of α7-nACh receptor by AEA was not reversed by either SR 141716A (Rimonabant), specific CB1 antagonist, or SR 144528, CB2 antagonist and was not sensitive to pertussis toxin treatment (Oz et al., 2003). In line with earlier findings, arachidonic acid (AA), a fatty acid moiety of AEA (Vijayaraghavan et al., 1995), but not ethanolamine or glycerol, inhibited the function of α7-nACh receptors suggesting that it was the intact endocannabinoid and not the metabolite AA that altered the function of α7-nACh receptor. In an *in vivo* study, Baranowska et al., demonstrated that, in the presence of AM2521, a CB1 antagonist, methanandamide, a metabolically stable analog of AEA inhibited nicotine-induced tachycardiac responses mediated by the activation of α7-nACh receptors on the cardiac postganglionic sympathetic neurons of urethane anesthetized rats (Baranowska et al., 2008). In another *in vivo* study, increased endocannabinoid levels modulate the somatic signs of nicotine withdrawal by a mechanism that does not involve CB1 receptors (Merritt et al., 2008).

Effects of synthetic cannabinoids on the functions of nACh receptors have been tested in a few studies. In *Xenopus* oocytes, WIN55,212-2 was ineffective up to concentrations of 10 μM, but CP55940 inhibited α7-nACh receptor with an IC_50_ value of 2.7 µM (Oz et al., 2004b). On the other hand, in cultured rat trigeminal ganglion neurons, patch-clamp studies indicated that native nicotinic receptor was inhibited by WIN55,212-2 with an IC_50_ value about 3 µM in a cannabinoid receptor independent manner (Lu et al., 2011).

Among the phytocannabinoids tested, THC and cannabinol up to 10 µM were ineffective (10-20% inhibition) on α7-nACh receptors expressed in *Xenopus* oocytes (Oz et al., 2004b; Mahgoub et al., 2013). Similarly, THC (1 µM) did not affect the amplitudes of currents through α4β2-nACh receptors (Spivak et al., 2007). CBD, on the other hand, at relatively high concentration modulates the function of nACh receptors. In earlier studies, CBD was reported to decrease the amplitudes of miniature end-plate potentials in frog neuromuscular junction suggesting an effect on the postsynaptic nACh receptors (Turkanis and Karler, 1986). Later studies indicated that CBD inhibits human α7-nACh receptors with an IC_50_ of 11.3 µM (Mahgoub et al., 2013; Oz et al., 2014) and suppresses choline-induced inward currents in rat hippocampal interneurons. CBD has also been shown to inhibit nicotine-induced [^3^H]-norepinephrine release in rat hippocampal slices (Mahgoub et al., 2013), and reduce withdrawal symptoms in nicotine-dependent rats (Smith et al., 2021) further suggesting an interaction between CBD and nicotinic receptors. Notably, Inhibitory effect of CBD was time-dependent with 50% inhibition occurring in 2.1 min sustained CBD application, voltage-independent, and non-competitive, i.e., while the EC_50_ of ACh remained unaltered, maximal ACh responses were significantly decreased (Mahgoub et al., 2013). In line with these findings, specific binding of [^125^I] α-bungarotoxin binding was not affected by CBD.

In addition to α7-nicotinic receptor, the α4β2 is the major nACh receptor subtype in the CNS and has been implicated in mediating both the positive-reinforcing and cognitive effects of nicotine (Tapper et al., 2004). The presence of the α4β2-nACh receptor subunit appears to be necessary and sufficient for the development of nicotine-induced tolerance and sensitization *in vivo* (Tapper et al., 2004). Using the whole-cell patch-clamp technique, Spivak et al., showed that AEA, in the concentration range of 200 nM to 2 μM, reduced the maximal amplitudes and increased the desensitization of acetylcholine-induced currents mediated by human α4β2 nACh receptors expressed in SH-EP1 cells (Spivak et al., 2007). The effects of AEA could be neither reversed by the SR141716A (1 μM) nor replicated by the THC (1 μM). Interestingly, AEA exerted inhibitory effect when applied extracellularly but not during intracellular dialysis. Kinetic analysis of the effect of AEA on α4β2 nACh receptor-mediated currents demonstrated that the first forward rate constant leading to desensitization increased nearly 30-fold as a linear function of AEA concentration and the energy levels of the activated state were raised by AEA (Spivak et al., 2007). Another study on α4β2 nACh receptors utilized rat thalamic synaptosomes where the ACh-induced ^86^Rb^+^ effluxes carried mainly through native α4β2 nACh receptors were reversibly inhibited by AEA with an IC_50_ of 0.9 µM in a noncompetitive manner (Butt et al., 2008). Inhibitory effect of AEA was not altered by pretreatments with the CB1 receptor antagonist SR141716A (1 μM), the CB2 receptor antagonist SR144528 (1 μM), and PTX. Although α7 and α4β2 receptor subtypes are affected by AEA, the function of muscle type nicotinic receptor is not altered by AEA (Oz, 2006). The results of key studies investigating the effects of cannabinoids on nACh receptors are summarized in Table 1.

**TABLE 1:** Effects of cannabinoids on nicotinic acetylcholine receptors.

Cannabinoid tested	Effect and conclusion	References
CBD	Decrease of the amplitudes of miniature end-plate potentials in frog neuromuscular junction	Turkanis and Karler, (1986)
AEA	Inhibition of α7 nACh receptors with IC_50_ of 229 nM in *Xenopus* oocytes	Oz et al. (2003)
2-AG	Inhibition of α7 nACh receptors with IC_50_ of 168 nM in *Xenopus* oocytes	Oz et al. (2004b)
CP55940	Inhibited α7-nACh receptors with an IC_50_ value of 2.7 µM in *Xenopus* oocytes	Oz et al. (2004b)
AEA	In the concentration range of 200 nM to 2 μM, reduced the maximal amplitudes and increased the desensitization of human α4β2 nACh receptors expressed in SH-EP1 cells	Spivak et al. (2007)
Methanandamide	Methanandamide (3 μmol/kg) produced an AM 251-insensitive inhibition of the nicotine-induced tachycardia	Baranowska et al. (2008)
AEA	Inhibited ACh-induced 86Rb^+^ effluxes with an IC_50_ of 0.9 µM in rat thalamic synaptosomes	Butt et al. (2008)
WIN55,212-2	Inhibited native nicotinic receptor with an IC_50_ value about 3 µM in cultured rat trigeminal ganglion neurons	Lu et al. (2011)
CBD	Inhibits human α7-nACh receptor with an IC_50_ of 11.3 µM in *Xenopus* oocytes and rat hippocampal interneurons	Mahgoub et al. (2013)

### Effects of cannabinoids on serotonin type 3 (5-HT_3_) receptors.

There are seven classes of 5-HT receptors (5-HT1–5-HT7). 5-HT_3_ receptor is the only ionotropic cation-selective ion channel which belongs to the superfamily of Cys-loop ligand-gated ion channels (Barnes et al., 2009; Lummis, 2012), remaining 5-HT receptor classes are G-protein-coupled receptors. To date, there are five 5-HT_3_ receptor genes termed A-E. Only 5-HT_3A_ subunit can assemble as a functional homo-pentameric channel and 5-HT_3A_ is an obligate participant in all other 5-HT_3_ receptor complexes (Lummis, 2012). Activation of 5-HT_3_ receptors depolarizes neurons and mediates fast, excitatory synaptic transmission in the central and peripheral nervous systems. The highest number of 5-HT_3_ receptor binding sites occurs in the area postrema and solitary tract nucleus (Morales and Wang, 2002). In addition, large numbers of 5-HT_3_ receptors are found in the gastrointestinal system where activation of 5-HT_3_ receptors are shown to alter gastrointestinal motility and to regulate the vomiting reflex (Sanger and Andrews, 2006), and they also appear to play a role in irritable bowel syndrome, visceral pain and inflammation (Faerber et al., 2007; Thompson and Lummis, 2007; Machu, 2011).

Effects of cannabinoids on the functions of 5-HT_3_ receptors were first demonstrated in nodose ganglion neurons (NGNs) and *Xenopus* oocytes (Fan, 1995; Oz et al., 1995). In NGNs, AEA, WIN55,212-12, CP55,940 and its nonpsychoactive enantiomer CP56,667 inhibited 5-HT_3_ receptors with IC_50_ values of 94 nM, 190 nM, 310 nM and 1.6 μM, respectively (Fan, 1995). Subsequent studies on 5-HT_3_ receptors expressed in *Xenopus* oocytes showed that AEA directly inhibits the function of 5-HT_3_ receptors with an IC_50_ value of 3.7 μM (Oz et al., 2002a). The inhibition developed gradually, reaching steady-state within 10–20 min, and it was non-competitive with respect to 5-HT. In both NGNs and *Xenopus* oocytes, agents known to modulate G-protein functions such as PTX treatments and intracellular applications of GDP-β-S, the nonhydrolyzable analogue of GTP, as well as applications of agents known to modulate intracellular cAMP levels did not alter the extent of AEA inhibition of 5-HT_3_ receptors (Fan, 1995; Oz et al., 2002a). In another study, using patch clamp technique in excised outside-out patch mode, it was demonstrated that cannabinoid receptor agonists THC, WIN55,212-2, AEA, JWH-015, LY320135, and CP55,940 inhibited the function of 5-HT_3_ receptors expressed in HEK-293 cells with IC_50_ values of 38, 104, 130, 147, 523, and 648 nM, respectively (Barann et al., 2002). Similarly, inhibition of 5-HT_3_ receptor mediated currents in rat trigeminal ganglion neurons by WIN55,212-2 was time and concentration-dependent (IC_50_ = 0.1 µM), and not reversed by cannabinoid receptor antagonists (Shi et al., 2012). The inhibition of 5-HT_3_ receptors by WIN55,212-2 in HEK-293 cells (Barann et al., 2002) and AEA in *Xenopus* oocytes (Oz et al., 2002a) was not altered by SR141716A, and their effects were noncompetitive. The cannabinoid receptor ligands [^3^H]-SR141716A and [^3^H]-CP55,940 did not specifically bind to parental HEK-293 cells. In competition experiments on membranes of HEK-293 cells transfected with the 5-HT_3_ receptor cDNA, WIN55,212-2, CP55,940, AEA and SR141716A did not affect the binding of [^3^H]-GR65630, a specific ligand for 5-HT3 receptors (Barann et al., 2002).

Interestingly, potencies of AEA actions on 5-HT_3_ receptors differ significantly between cell lines such as HEK-293 (Barann et al., 2002) and *Xenopus* oocytes (Oz et al., 2002a). The mechanisms underlying these seemingly discrepant results were studied in a recent study (Xiong et al., 2008). It was found that differences in the potencies of AEA inhibition of 5-HT_3_ receptors between *Xenopus* laevis oocytes and HEK-293 cells were mainly due to different levels of steady-state receptor density at the cell surface. The magnitude of AEA inhibition was inversely correlated with the expression level of receptor protein; i.e., increasing surface receptor expression decreased the magnitude of AEA inhibition. In line with these findings, pretreatment with actinomycin D, an inhibitor of transcription process, decreased the amplitude of current activated by maximal 5-HT concentrations and increased the magnitude of AEA inhibition. AEA accelerated 5-HT_3_ receptor desensitization time in a concentration-dependent manner without significantly changing receptor activation and deactivation time. The desensitization time was correlated with the AEA-induced inhibition and mean 5-HT current density. Applications of 5-hydroxyindole and nocodazole, a microtubule disruptor, significantly slowed 5-HT_3_ receptor desensitization and reduced the magnitude of AEA inhibition. Collectively, these findings suggested that 5-HT_3_ receptor density at the steady state regulates receptor desensitization kinetics and the potency of AEA-induced inhibiting effect on the receptors. Thus, it appears that the differences in IC_50_ values for AEA inhibition of 5-HT_3_ receptor in *Xenopus* oocytes (3.7 μM; (Oz et al., 2002a); and HEK-293 cells (130 nM; (Barann et al., 2002)) are mainly due to the different receptor expression levels among various cell types.

In *vivo* studies, WIN55,212-2 and CP55,940 inhibited the responses mediated by 5-HT_3_ receptors located on the terminals of cardiopulmonary afferent C-fibers in anesthetized and SR141716A pretreated rats (Godlewski et al., 2003). Similarly, CBD inhibited the reflex bradycardia induced by the 5-HT_3_ receptor agonist phenylbiguanide in spontaneously hypertensive rats (Kossakowski et al., 2019). In another *in vivo* study, the inhibitory effect of WIN55,212-2 on cocaine-induced locomotor hyperactivity was reported to be mediated by the inhibition of 5-HT_3_ receptors (Przegaliński et al., 2005). In a recent study, mice deficient in CB1 and CB2 receptors were treated with THC and AEA, in the presence of the 5-HT_3_ antagonist ondansetron (Rácz et al., 2008). AEA induced analgesia, but not catalepsy, is completely blocked by ondansetron, suggesting that 5-HT_3_ receptors are involved in cannabinoid-induced analgesia in a manner independent of known cannabinoid receptors (Rácz et al., 2008).

Both THC and CBD were shown to directly interact with 5-HT_3_ receptors expressed in HEK-293 cells (Barann et al., 2002; Xiong et al., 2011b), *Xenopus* oocytes, and NGNs (Yang et al., 2010a; Yang et al., 2010b; Xiong et al., 2011b). Depending on the expression levels of the receptor, THC and CBD inhibited human 5-HT_3A_ receptors with respective IC_50_ values of 1.2 µM and 0.6 µM in *Xenopus* oocytes (Yang et al., 2010a; Yang et al., 2010b) and 119 nM and 329 nM in HEK-293 cells (Xiong et al., 2011b). Inhibition by these cannabinoids was voltage-independent, non-competitive, and increased with sustained application (preincubation) time. In line with non-competitive nature of their effect, THC and CBD did not alter the specific [^3^H]GR65630 binding on 5-HT_3A_ receptors (Yang et al., 2010a; Yang et al., 2010b). Importantly, slowing the receptor desensitization by pharmacological agents such as 5-hydroxyindole and nocodazole or mutations such as R427L markedly decreased the extent of 5-HT_3_ receptor inhibition by CBD (Xiong et al., 2011b). Similarly, increasing the expression level and density of 5-HT_3_ receptors on the cell membrane caused a noticeable decrease of receptor desensitization and the potency of THC on 5-HT_3_ receptor (Yang et al., 2010b). Finally, 5-HT3 and CB1 receptors colocalize in hippocampal and dentate gyrus interneurons (Morales and Bäckman, 2002) and in other brain regions as well (Morales et al., 2004). Therefore, it is likely that the actions of cannabinoids including AEA and THC within the same cell can be mediated by simultaneous dual actions of cannabinoids on cannabinoid and 5-HT_3_ receptors. The results of key studies investigating the effects of cannabinoids on 5-HT_3_ receptors are summarized in Table 2.

**TABLE 2:** Effects of cannabinoids on 5-HT_3_ receptors.

Cannabinoid tested	Effect and conclusion	References
AEA	Inhibited 5-HT_3_ receptors with IC_50_ value of 94 nM in nodose ganglion neurons	Fan, (1995)
WIN55,212-12 and CP55,940	WIN55,212-12 and CP55,940 inhibited 5-HT_3_ receptors with IC_50_ values of 310 nM and 1.6 μM, respectively in nodose ganglion neurons	Fan, (1995)
AEA	Inhibits 5-HT_3_ receptors with an IC_50_ value of 3.7 μM in *Xenopus* oocytes	Oz et al. (2002a)
THC	Inhibits 5-HT_3_ receptors with an IC_50_ value of 38 nM in HEK-293 cells	Barann et al. (2002)
AEA	Inhibits 5-HT_3_ receptors with an IC_50_ value of 130 nM in HEK-293 cells	Barann et al. (2002)
WIN55,212-2, JWH-015, LY320135, and CP55940	WIN55,212-2, JWH-015, LY320135, and CP55940 inhibit 5-HT_3_ receptors with IC_50_ values of 104, 147, 523, and 648 nM, respectively in HEK-293 cells	Barann et al. (2002)
WIN55,212-2 and CP55,940	They inhibited the cardiovascular responses mediated by 5-HT_3_ receptors in anesthetized and SR141716A pretreated rats	Godlewski et al. (2003)
AEA	AEA induced analgesia is completely blocked by ondansetron in CB1 and CB2 knock-out mice	Rácz et al. (2008)
CBD	Inhibited human 5-HT_3A_ receptors with an IC_50_ of 0.6 µM in *Xenopus* oocytes	Yang et al. (2010a)
THC	Inhibited human 5-HT_3A_ receptors with an IC_50_ of 1.2 µM in *Xenopus* oocytes	Yang et al. (2010b)
CBD	Inhibited human 5-HT_3A_ receptors with an IC_50_ of 329 nM in HEK-293 cells	Xiong et al. (2011b)
THC	Inhibited human 5-HT_3A_ receptors with an IC_50_ of 119 nM in HEK-293 cells	Xiong et al. (2011b)
WIN55,212-2	Inhibition of 5-HT_3_ receptor with an IC_50_ of 0.1 µM in rat trigeminal ganglion	Shi et al. (2012)
CBD	Inhibited the reflex bradycardia induced by the 5-HT3 receptor agonist phenylbiguanide in spontaneously hypertensive rats	Kossakowski et al. (2019)

### Effects of cannabinoids on ionotropic glutamate receptors

Glutamate mediates most excitatory neurotransmission in the mammalian central nervous system through activation of metabotropic, G protein–coupled glutamate receptors, and ionotropic glutamate receptors, which are cation-selective ligand-gated ion channels. Ionotropic glutamate receptors are divided into different functional classes, namely α-amino-3-hydroxy-5-methyl-4-isoxazolepropionic acid (AMPA) receptors, kainate receptors, N-methyl-D-aspartate (NMDA) receptors, and GluD (Δ) receptors (Hansen et al., 2021). In *vivo* studies, synthetic cannabinoid HU211 protected against tremorogenic, convulsive, and lethal effects of NMDA in mice and acted as a functional antagonist in radioligand binding studies (Feigenbaum et al., 1989). Similarly, NMDA-induced toxicity was reversed by THC, but not WIN55,212-2 in SR141716A insensitive manner in AF5 cells (Chen et al., 2005). AEA (1-10 µM), but not THC, potentiated NR1/NR2A NMDA receptor-mediated currents in *Xenopus* oocytes and increased NMDA-induced intracellular Ca^2+^ transients in the presence of SR141716A in rat cortical, cerebellar, and hippocampal slices (Hampson et al., 1998). 2-AG (5 µM) enhanced NMDA-evoked currents in a CB1 and TRPV1 receptor-independent manner through activation of PKC/Src signaling pathway (Yang et al., 2014). In studies of blood pressure control in rats, it was found that AEA produces a pressor effect in the presence of SR141716A and this increase in pressure was partially reduced by the NMDA receptor antagonist MK-801, suggesting a possible direct interaction between NMDA receptors and AEA with relevance to central control of blood pressure (Malinowska et al., 2010).

CBD inhibited AMPA-glutamate receptor-mediated evoked excitatory postsynaptic currents and suppressed the frequency and amplitudes of miniature excitatory postsynaptic currents in mice hippocampal neurons (Yu et al., 2020). Furthermore, in HEK-293 cells, CBD was shown to inhibit recombinant GluA1 currents with IC_50_ of 22.5 µM and facilitate the deactivations of GluA1 and GluA2 receptors and slow the recovery of GluA1 receptor from desensitization (Yu et al., 2020). In line with these findings, CBD alleviate behavioral hyperactivity and hippocampal c-Fos expression in Gria1−/− mice which is a model sensitive to drugs that downregulate glutamatergic transmission such AMPA antagonists (Aitta-Aho et al., 2019). AEA, at high concentrations, inhibits homomeric GluR1 and GluR3 subunit-mediated currents with IC_50_ of 161 µM and 143 μM, respectively (Akinshola et al., 1999). AEA inhibited heteromeric GluR1/3 and GluR2/3 receptor subunits with similar IC_50_ value of 148 µM and 241 μM, respectively.

### Effects of cannabinoids on glycine receptors

Glycine receptors are inhibitory, anion-selective Cys-loop ligand-gated ion channels that mediate fast inhibitory neurotransmission in the spinal cord, brainstem and retina (Lynch, 2009). There are four known glycine receptor α subunits (α1–α4) and a single β subunit in vertebrates. Functional receptors assemble as homo-pentamers of α subunits, or as hetero-pentamers of α and β subunits (Burgos et al., 2016). Malfunctions of glycine receptors have been associated with a range of neurological disorders including hyperekplexia, temporal lobe epilepsy, autism, breathing disorders, and chronic inflammatory pain (Lynch et al., 2017).

In earlier studies, co-application of glycine with AEA or 2-AG (0.2-2 µM), independent of activation of CB1 and TRPV1 receptors, markedly inhibited peak amplitudes and accelerated onset and desensitization of high (100 µM) glycine-activated currents in pyramidal neurons isolated from the hippocampus of neonatal rats (Lozovaya et al., 2005). In contrast to endocannabinoids, WIN55,212-2 (1 µM) did not significantly affect peak amplitudes but significantly accelerated the desensitization as well as onset of currents activated by high concentrations (100 µM) of glycine (Lozovaya et al., 2005; Yatsenko and Lozovaya, 2007) but potentiated the amplitudes of currents at low (40 µM) glycine concentrations (Iatsenko et al., 2007). Other cannabinoids such as HU-210 (Yang et al., 2008; Demir et al., 2009; Xiong et al., 2012b) and ajulemic acid (Ahrens et al., 2009b) also act as positive allosteric modulators of α1 glycine receptors.

Co-application of AEA (1-10 µM) with low (EC_10_; 3-10 µM) concentrations of glycine potentiated the glycine-induced currents mediated by homomeric α1, α2, and α3 glycine receptors expressed in HEK-293 cells (Yang et al., 2008; Yévenes and Zeilhofer, 2011), *Xenopus* oocytes, and CNS neurons (Hejazi et al., 2006) in a CB receptor-independent manner. AEA-induced potentiation of these three glycine receptor subtypes was reduced by mutating a conserved intracellular lysine residue (K385A), whereas potentiation of α1 receptor induced by N-arachidonoyl-glycine was reduced by mutating loop 2 (A52), transmembrane 2 (G254) or intracellular (K385) amino acids (Yévenes and Zeilhofer, 2011). In another study, AEA (1-30 µM) substantially increased the amplitude of glycine-activated currents in cultured rat spinal neurons (Xiong et al., 2012b). The effect reached a steady-state within 5 min continuous AEA application and markedly diminished by increasing glycine concentrations. In HEK-293 cells, AEA potentiation of homomeric α1 and α3 receptors was significantly higher than α2 glycine receptors. While S296A and S307A mutations in transmembrane three domains of α1 and α3 receptors markedly decreased the potentiation by sustained (continuous), but not simultaneous co-application of AEA, A303S mutation increased the potentiation of α2 glycine receptors by AEA. The S296A mutation also decreased the extent of α1 receptor potentiation by other cannabinoids such as CBD, THC, and HU-210 (Xiong et al., 2012b). Importantly, the authors of this study also showed in control studies that these mutations have no significant effect on the pharmacological properties of glycine receptors. Furthermore, removal of hydroxy or oxygen groups in AEA and THC significantly reduced the potentiating effects of these compounds on α1 glycine receptor suggesting that these groups are important for their effects on glycine receptor. In outside-out patches in CHO cells expressing human α1 glycine receptor, continuous application of 2-AG (1 µM) inhibited the peak amplitudes, decreased the rise time, accelerated the desensitization rate, and slowed down the deactivation of the receptor activated by glycine at the level of EC_50_ (40-70 µM). Notably, inhibitory effect of AEA was significantly less at low (20 µM) and high (1 mM) concentrations of glycine (Lozovaya et al., 2011). In further studies mimicking synaptic stimulation in outside-out patches from CHO cells by applying high concentration of glycine (1 mM) for 2 ms duration at 10 Hz frequency, application of 2-AG markedly reduced the amplitudes of glycine-induced currents. Similarly, in hypoglossal motoneurons of CB1 knock-out mice, 2-AG abolished the facilitation of synaptic glycinergic currents induced by repetitive (10-20 Hz) stimulations indicating that endocannabinoids can modulate synaptic transmission under physiological conditions in a cannabinoid-receptor-independent manner (Lozovaya et al., 2011).

CBD and THC have been shown to potentiate glycine receptor-mediated currents in a cannabinoid receptor-independent manner in *Xenopus* oocytes expressing homomeric α1 and heteromeric α1β glycine receptors, and in acutely isolated CNS neurons (Hejazi et al., 2006; Xiong et al., 2011a; Wells et al., 2015). CBD was reported to act as positive allosteric modulator of α1 and α1β1 glycine receptors expressed in HEK-293 cells with EC_50_ values of 12.3 µM and 18.1 µM, respectively (Ahrens et al., 2009a). Notably, at higher concentration range, CBD directly acts as agonist of α1 and α1β1 glycine receptors with respective EC_50_ values of 132.4 µM and 144.3 µM. The mutation of the α1 subunit transmembrane two serine 267) residue to isoleucine abolished both co-activation and direct activation of glycine receptor by CBD (Foadi et al., 2010). However, THC-induced potentiation was not affected when the S267Q mutant α1 glycine receptors were expressed in *Xenopus* oocytes (Hejazi et al., 2006). In subsequent studies, it was shown that continuous application, but not co-application, of THC (1 µM) for about 5 min caused a gradually developing marked potentiation (up to 1,200%) of glycine activated currents in HEK-293 cells expressing α1, α3, and α1β1, but not α2, subunits of glycine receptor (Xiong et al., 2011a). Notably, S296 residue of the α1 and the S307 of the α3 subunits have been shown to be critical for the potentiation of glycine receptors by continuous, but not simultaneous, THC application (Xiong et al., 2011a; Xiong et al., 2012b). Conversely, substitution of the corresponding residue, Ala303 of the α2 subunit with a serine converted the α2 subunit from a low to high THC sensitivity. Similar to THC, the CBD, dehydroxyl-CBD, and dehydroxyl-THC have also been shown to potentiate glycine-activated currents of α1 and α3 subunits (Xiong et al., 2011a; Xiong et al., 2012a; Xiong et al., 2012b). Further studies indicated that CBD and dehydroxyl-CBD suppress persistent inflammatory and neuropathic pain by potentiating α3 glycine receptor-mediated currents in HEK-293 cells and cultured spinal neurons and the CBD suppression of neuropathic pain was reversed in α3-knock-out mouse (Xiong et al., 2012a). In subsequent studies, dehydroxyl-CBD was reported to suppress inflammatory pain and potentiate α1-glycine receptors by interacting with S296 amino acid residue. Accordingly, analgesic effect of dehydroxyl-CBD was reversed in the transgenic mice carrying S296A mutation, which also blocks DH-CBD-induced potentiation of glycine-activated currents in neurons of the spinal cord dorsal horn in mutant mouse (Lu et al., 2018). In another study, dehydroxyl-CBD rescued functional deficiency of glycine receptor and exaggerated acoustic and tactile startle responses in mice bearing point mutations in α1 glycine receptors that are responsible for a hereditary startle-hyperekplexia disease (Xiong et al., 2014). Recently, cannabinoids such as THC and CBD were reported to rescue cocaine-induced seizures by restoring brain glycine receptor dysfunction in a cannabinoid-receptor independent manner (Zou et al., 2020b). The results of key studies investigating the effects of cannabinoids on glycine receptors are summarized in Table 3.

**TABLE 3:** Effects of cannabinoids on glycine receptors.

Cannabinoid tested	Effect and conclusion	References
AEA	Inhibition of peak amplitudes and increasing desensitization of glycine induced currents by AEA (0.2-2 µM) in pyramidal neurons of neonatal rat hippocampus	Lozovaya et al. (2005)
2-AG	Inhibition of peak amplitudes and increasing desensitization of glycine induced currents by 2-AG (0.2-2 µM) in pyramidal neurons of neonatal rat hippocampus	Lozovaya et al. (2005)
AEA	Potentiation of glycine (low concentrations)-induced currents mediated by α1 and α1β1 receptors with EC50 of 319 and, 318 nM, respectively, in *Xenopus* oocytes and also in ventral tegmental area neurons	Hejazi et al. (2006)
THC	Potentiation of glycine (low concentrations)-induced currents mediated by α1 and α1β1 receptors with EC_50_ of 86 and 71 nM, respectively, in *Xenopus* oocytes and also in ventral tegmental area neurons	Hejazi et al. (2006)
WIN55,212-2	Potentiation of glycine (low concentrations) currents in pyramidal neurons of rat hippocampus	Iatsenko et al. (2007)
AEA	Potentiated the glycine (low concentration)-induced currents mediated by homomeric α1, α2, and α3 glycine receptors expressed in HEK-293 cells	Yang et al. (2008)
HU-210	Potentiation of α1 and α1β but inhibition of α2 subunit currents in HEK-293 cells	Yang et al. (2008)
WIN55,212-2	Inhibition of α2 and α3 subunit currents in HEK-293 cells	Yang et al. (2008)
HU-210	Potentiation of α1subunit currents with an EC_50_ of 5.1 µM in HEK-293 cells	Demir et al. (2009)
CBD	Potentiated α1 and α1β1 glycine receptors expressed in HEK-293 cells with EC_50_ values of 12.3 and 18.1 µM, respectively	Ahrens et al. (2009a)
Ajulemic acid	Potentiation of α1 and α1β subunit glycine receptors in HEK-293 cells with EC_50_ values of 9.7 and 12.4 μM, respectively	Ahrens et al. (2009b)
2-AG	Inhibited α1 subunit glycine receptors expressed in CHO cells and in hypoglossal motoneurons of CB1 knock-out mice	Lozovaya et al. (2011)
N-arachidonoyl-glycine	Potentiated the glycine (low concentration)-induced currents mediated by α1, but inhibited α2, and α3 glycine receptors expressed in HEK-293 cells	Yévenes and Zeilhofer, (2011)
AEA	Potentiated the glycine (low concentration)-induced currents mediated by homomeric α1, α2, and α3 glycine receptors expressed in HEK-293 cells	Yévenes and Zeilhofer, (2011)
THC	Potentiation of glycine activated currents in HEK-293 cells expressing α1, α3, and α1β1, but not α2, subunits of glycine receptor	Xiong et al. (2011a)
CBD	Potentiate glycine-activated currents of α3 subunits expressed in HEK-293 cells and dorsal horn neurons in rat spinal cord slices	Xiong et al. (2012a)
AEA	Potentiated the glycine-activated currents mediated by homomeric α1, α2, and α3 glycine receptors expressed in HEK-293 cells and in cultured rat spinal neurons	Xiong et al. (2012b)
CBD and THC	Potentiated the low glycine-activated currents mediated by homomeric α1 glycine receptors expressed in HEK-293 cells	Xiong et al. (2012b)

### Effects of cannabinoids on GABA_A_ receptors

γ-Aminobutyric acid (GABA), the major inhibitory neurotransmitter in the brain, exerts its action *via* ionotropic GABA_A_ and metabotropic GABA_B_ receptors. GABA_A_ receptors, like glycine receptors, are anion-selective Cys-loop ligand-gated ion channels activated by GABA and the selective agonist muscimol, blocked by bicuculline and picrotoxin, and modulated by benzodiazepines, barbiturates, and some other classes of depressants (Olsen and Sieghart, 2008; Sigel and Steinmann, 2012). Consequently, drugs modulating the activity of GABA_A_ receptors play a critical role in the treatment of neuropsychiatric disorders such as epilepsy, anxiety, and insomnia, as well as in anesthesia (Olsen and Sieghart, 2008; Olsen, 2018; Ghit et al., 2021). A total of 19 GABA_A_ receptor subunit genes have been identified in humans that code for six α1-6, three β1-3, three γ1-3, three ρ1-3, and one each of the δ, ε, π, and θ (Sigel and Steinmann, 2012). They are usually constructed with two copies of an α subunit, two copies of a β subunit, and one copy of either a γ subunit, or another such as Δ, to form several combinations of hetero-pentameric GABA_A_ receptor subtypes (Sigel and Steinmann, 2012; Olsen, 2018).

In early radioligand binding studies, synthetic cannabinoids such as levonantradol, nabilone, CP-47,497 (-)-CP-55,940 and (-)-CP-55,244 enhanced the specific binding of [^3^H]-flunitrazepam to mouse brain *in vivo*, potentiated the anticonvulsant effects of diazepam against pentylenetetrazol, and elicited analgesic effects that correlated with the potency of their facilitatory effects on [^3^H]-flunitrazepam binding (Koe and Weissman, 1981; Koe et al., 1985) suggesting that cannabinoids can interact with GABA_A_ receptors. Furthermore, benzodiazepines such as diazepam partially substitutes for THC in drug discrimination studies (Mokler et al., 1986) and this effect was not antagonized by CB1 receptor antagonist SR141716 (Wiley and Martin, 1999). More recently, effects of cannabinoids on the functional properties of GABA_A_ receptors has been shown in heterologous expression systems and native neurons. In *Xenopu*s oocytes expressing α1-6β2γ2 subunits of GABA_A_ receptors, 2-AG potentiated currents activated low GABA concentrations (EC_5_ = 0.1-10 µM) with EC_50_ values ranging from 1.5 µM to 15.7 µM and significantly shifted GABA concentration-response curves to the left (Sigel et al., 2011; Bakas et al., 2017). 2-AG also potentiated extrasynaptic δ-subunit containing α4β2δ GABA_A_ receptors (Bakas et al., 2017). Potentiation of GABA-evoked currents by 2-AG was significantly greater at GABA_A_-receptors containing a β2 or β3 subunit and markedly reduced by α2β2(V436T)γ2L and α2β2(VF439L)γ2L mutations in transmembrane domain 4 (Sigel et al., 2011). Subsequent studies have identified four amino acid residues in transmembrane domain 4 of the β2 subunit, β2W428, β2S429, β2F432, and β2V443 and in transmembrane domain 3, β2V302, in addition to the residues previously described, β2M294 and β2L301 (Baur et al., 2013b). In addition to cannabinoid receptor agonists, antagonists of these receptors have also been shown to directly modulate GABA_A_ receptors. SR141716 (rimonabant) and AM251 allosterically potentiated low (0.5 µM) GABA activated currents to maximal potentiations of 3,381% and 881% of controls with EC_50_ values of 7.3 µM and 0.4 µM, respectively in *Xenopus* oocytes expressing α1β2γ2 GABA_A_ receptor (Baur et al., 2012). Another study on dissociated basolateral amygdala neurons, reported that AM251 (1 µM) caused 30% reduction on the peak amplitudes of GABA (5 µM)-activated currents (Zhu and Lovinger, 2005).

In HEK-293 cells expressing α1β2γ2 and α2β2γ2 subunit combinations of GABA receptors and acutely isolated hippocampal pyramidal neurons from rat brain, 2-AG, AEA, and CP55,940, at 1 µM concentrations, significantly inhibited peak amplitudes and increased desensitization of currents activated by high (1 mM) concentration of GABA (Golovko et al., 2015). In CB1 knock-out mice or in rat somatosensory cortex pyramidal neurons pretreated with 5 μM SR141716A, synthetic cannabinoids CP55,940 (1 µM) and WIN55,212-2 (5 µM) gradually reduced the amplitudes of evoked GABAergic postsynaptic currents.

CBD has been shown to act as a positive allosteric modulator, with EC_50_ values ranging from 0.9 µM to 16.1 µM and magnitudes of potentiation in the range of 72%–332% at αβγ2 receptor combinations, with higher level of potentiation on α2 containing subtype combination (Bakas et al., 2017). The greatest levels of enhancements by CBD were reported as 332% on α2β2γ2L and 752% on α4β2δ subunit combinations and the classical benzodiazepine binding site located at α-γ2L interface does not seem to be involved in CBD interaction with GABA_A_ receptors. Furthermore, CBD potentiation of α2β2γ2L was significantly decreased by α2β2(V436T)γ2L mutation on transmembrane domain 4. Notably, CBD potentiation of GABA_A_ receptors was significantly decreased with increasing GABA concentrations, resulting in decreased GABA EC_50_ values with no apparent change in the E_max_. In a recent study, CBD (2 µM) induced 30-50% potentiation of GABA (1-10 µM) activated currents in *Xenopus* oocytes expressing human α1β2γ2, α1β2, and α2β2γ2, α2β2 subunit combinations of GABA_A_ receptors (Ruffolo et al., 2018). In another investigation, CBD (10 µM) caused 106% potentiation, with EC_50_ of 2.4 µM, of GABA (15 µM)-activated currents in *Xenopus* oocytes expressing α1β2γ2 subunit combination of GABA_A_ receptors (Anderson et al., 2019). In addition to CBD, THC (3 µM) has also been shown to potentiate (about 100%) α1β2γ2 GABA_A_ receptors activated by low concentrations (EC_2_) of GABA (Yao et al., 2020b). In another study, the run-down of GABA (500 µM) activated currents in *Xenopus* oocytes transplanted with hippocampal membranes from epileptic patients was significantly reversed by 2 h pretreatment with 50 nM cannabidivarin, a non-psychoactive homolog of CBD (Morano et al., 2016). In a recent study, cannabigerolic acid, the biosynthetic precursor to both THC and CBD, has been shown to increase GABA (15 µM) activated currents to 271% of controls with EC_50_ of 910 nM in *Xenopus* oocytes expressing human α1β2γ2 GABA_A_ receptors (Anderson et al., 2021). In addition, this study showed that cannabigerolic acid potentiated the anticonvulsant effects of clobazam against hyperthermia-induced and spontaneous seizures and displayed anticonvulsant effects in maximal electroshock model. Recently, dehydroxylcannabidiol, a synthetic nonpsychoactive cannabinoid, has been shown to restore the GABA- and glycine-activated currents in HEK-293 cells co-expressing a major GABA_A_ receptor isoform (α1β2γ2) and α1 glycine receptor carrying a human hyperekplexia-associated mutation (R271Q), suggesting that cannabinoids can be potentially valuable candidate drugs to manage hyperekplexia (Zou et al., 2020a). Collectively, these results indicate that both CBD and THC act as positive allosteric modulators of glycine and GABA_A_ receptors, especially at low agonist concentrations and subunit specific manner. The results of key studies investigating the effects of cannabinoids on GABA_A_ receptors are summarized in Table 4.

**TABLE 4:** Effects of cannabinoids on GABA_A_ receptors.

Cannabinoid tested	Effect and conclusion	References
AM251	30% reduction on the peak amplitudes of GABA (5 µM)-activated currents by 1 µM AM251 in isolated basolateral amygdala neurons	Zhu and Lovinger, (2005)
2-AG	Potentiated α1-6β2γ2 subunits of GABA_A_ receptors activated by low GABA (0.1-10 µM) with EC_50_ values ranging from 1.5 µM to 15.7 µM in *Xenopus* oocytes	Sigel et al. (2011)
SR141716 and AM251	Potentiated low (0.5 µM) GABA activated currents with EC_50_ values of 7.3 and 0.4 µM, for SR141716 and AM251, respectively in *Xenopus* oocytes expressing α1β2γ2 GABA_A_ receptor	Baur et al. (2012)
AEA and 2-AG	Inhibited peak amplitudes and increased desensitization of currents activated by high (1 mM) GABA in HEK-293 cells expressing α1β2γ2 and α2β2γ2 subunits of GABA_A_ receptors and acutely isolated rat hippocampal pyramidal neurons	Golovko et al. (2015)
CP55,940	Inhibited peak amplitudes and increased desensitization of currents activated by high (1 mM) GABA in HEK-293 cells expressing α1β2γ2 and α2β2γ2 subunits of GABA_A_ receptors and acutely isolated rat hippocampal pyramidal neurons	Golovko et al. (2015)
2-AG	Potentiated α1-6β2γ2 and extrasynaptic δ-subunit containing α4β2δ GABA_A_ receptors at low GABA concentrations in *Xenopus* oocytes	Bakas et al. (2017)
CBD	Potentiated α1-6β2γ2 and extrasynaptic δ-subunit containing α4β2δ GABA_A_ receptors at low GABA concentrations with EC_50_ values ranging from 0.9 µM to 16.1 µM in *Xenopus* oocytes	Bakas et al. (2017)
CBD	Potentiation of GABA (1-10 µM) activated currents in *Xenopus* oocytes expressing human α1β2γ2, α1β2, and α2β2γ2, α2β2 subunit combinations of GABA_A_ receptors	Ruffolo et al. (2018)
CBD	Potentiation, with EC50 of 2.4 µM, of GABA (15 µM)-activated currents in *Xenopus* oocytes expressing α1β2γ2 subunit combination of GABAA receptors	Anderson et al. (2019)
THC	Potentiate α1β2γ2 GABA_A_ receptors activated by low concentrations of GABA	Yao et al. (2020b)
Cannabigerolic acid	Potentiate GABA activated currents with EC_50_ of 910 nM in *Xenopus* oocytes expressing human α1β2γ2 GABA_A_ receptors	Anderson et al. (2021)

## Discussion

Cannabinoids are highly lipophilic compounds with a LogP (octanol–water partition coefficient) values ranging between 4 and 9. Thus, it is likely that these lipophilic molecules first dissolve into the lipid membrane and then diffuse into a non-annular lipid space to allosterically inhibit the ion channels. Consistent with this idea, the effect of cannabinoids on ion channels usually reaches a maximal level within several minutes (5-10 min) of sustained applications.

**FIGURE 1:**
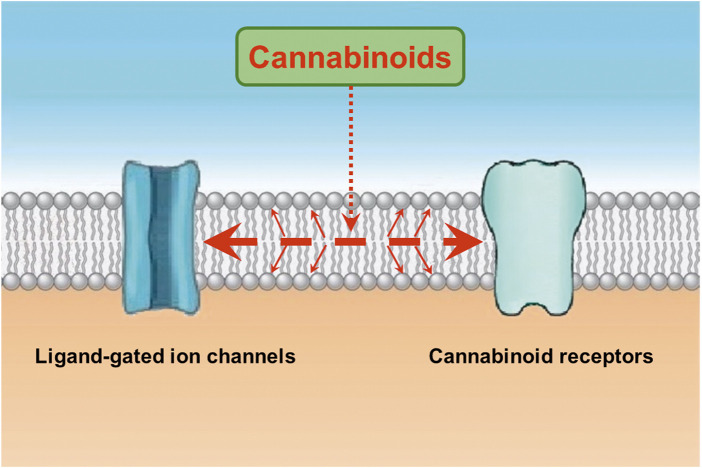
Cannabinoids interact with the lipid membrane and influence the functional properties of ion channels and other integral membrane proteins. Cannabinoids enter the lipid membrane and binds to cannabinoid receptors through lipid membrane (Reggio and Traore, 2000; Barnett-Norris et al., 2005; Makriyannis et al., 2005). In addition, cannabinoids directly affect channel function by changing the biophysical properties of the lipid membrane or binding to a hydrophobic binding site(s) located on the transmembrane regions of ligand-gated ion channels (represented with large dashed red arrow). Cannabinoids, like other lipophilic molecules, partition into the lipid bilayer and alter the biophysical properties of the membrane by reducing membrane electrical resistance, increasing membrane fluidity, changing membrane order, increasing membrane stiffness, increasing membrane elasticity, and changing physicochemical and structural properties of bilayer membranes (represented with thinner diagonal red arrows). Secondly, cannabinoids can bind directly to transmembrane domains of ion channels embedded in the cell membrane (see discussion).

Notably, functional properties of ligand-gated ion channels have been shown to be affected by the activation of second messenger pathways (Siara et al., 1990; Hoffman et al., 1994; Zhang et al., 1995; Nishizaki and Sumikawa, 1998). However, agents modulating cAMP and protein kinase C pathways and chelation of intracellular Ca^2+^ do not alter the effects of endocannabinoids on LGICs (Oz et al., 2002a; Oz et al., 2003). Beside cannabinoids, actions of several lipophilic modulators, such as capsaicin (Lundbaek et al., 2005; Alzaabi et al., 2019; Nebrisi et al., 2020), endocannabinoids (Oz et al., 2004a; Spivak et al., 2007), general anesthetics (Zhang et al., 1997; Jackson et al., 2008), and steroids (Oz et al., 2002b) on various ion channels require 5-10 min continuous application times to reach their maxima, suggesting that their binding site(s) is/are located inside the lipid membrane and require a relatively slow equilibrium time to exert their effect. From this aspect, it appears that alone or the combination of two mechanisms can describe the lipophilic actions of cannabinoids (Oz, 2006; Oz et al., 2014; Ghovanloo and Ruben, 2021). First, cannabinoids, like other lipophilic molecules, partition into the lipid bilayer and alter the biophysical properties of the membrane by reducing membrane electrical resistance (Bach et al., 1976), increasing membrane fluidity (Hillard et al., 1985; Mavromoustakos et al., 2001; Dainese et al., 2012), changing membrane order (Bloom et al., 1997), increasing membrane stiffness (Ghovanloo et al., 2021; Ghovanloo and Ruben, 2021), increasing membrane elasticity (Medeiros et al., 2017; James et al., 2022), and changing physicochemical and structural properties of bilayer membranes (Makriyannis et al., 1990; Yang et al., 1992; Ambrosi et al., 2005; Tiburu et al., 2007; Tian et al., 2011). Secondly, cannabinoids can bind directly to transmembrane domains of ion channels embedded in the cell membrane (Xiong et al., 2011a; Xiong et al., 2012b; Lu et al., 2018; Ghovanloo et al., 2021). In fact, residue S296 in transmembrane domain of α1 glycine receptor has been reported to interact with cannabinoids and mediate their potentiating effect on these receptors (Xiong et al., 2011a; Xiong et al., 2012b; Lu et al., 2018). In favor of specific binding site on ion channels, WIN55,212-3, cannabinoid receptor-inactive enantiomer of WIN55,212-2, did not affect the 5-HT_3_ receptor-mediated currents (Barann et al., 2002), confirming earlier results with CP56667 on nodose ganglion neurons (Fan, 1995) and indicating that although their effects are not mediated by the activation of cannabinoid receptors, these synthetic cannabinoids inhibit 5-HT_3_ receptors in an enantiomer-specific manner possibly through a hydrophobic bindings site within the bilayer membrane. As a result of these mechanisms, it is likely that cannabinoids affect the energy requirements for gating-related conformational changes and allosterically modulate the functional properties of ion channels (Spivak et al., 2007). Interaction between allosteric modulators and cannabinoids has also been investigated in a few studies. While, general anesthetics (Jackson et al., 2008), ethanol (Oz et al., 2005), and neurosteroids (Sigel et al., 2011) exert additive effects with endocannabinoids, fatty acid amides such as docosatetraenylethanolamide (Baur et al., 2013a; Baur et al., 2013b) antagonize the positive allosteric effects of endocannabinoids suggesting distinct binding sites for these modulators. In conclusion, both membrane disturbing effects and a hydrophobic bindings site(s) within the transmembrane regions of the LGIC can mediate the modulatory actions of cannabinoids on these channels (Figure 1).

Cannabinoids also appear to interact with other membrane lipids such as cholesterol to exert their effects (Martin et al., 2021). The presence of cholesterol in phosphatidylcholine bilayers increased THC- phosphatidylcholine complex formation and high cholesterol concentrations were proposed to enhance THC activity in the bilayer membrane (Bruggemann and Melchior, 1983). In another study, cholesterol was shown to stimulate both the insertion of AEA into bilayer membranes, and its transport across these membranes (Di Pasquale et al., 2009). Interestingly, cholesterol seems to provide structural support to CBD binding site on the glycine receptors. Removal of membrane cholesterol by cyclodextrin (Yao et al., 2020b) or anti-cholesterol drug such as simvastatin (Yao et al., 2020a) markedly reversed THC or dehydroxyl-CBD potentiation of α1 and α3 glycine receptors by interacting with the S296 residue suggesting that cholesterol directly interact with these cannabinoids on this binding pocket. Another recent study indicates that membrane orientation of CBD and its effect on water permeability were significantly altered in the presence of cholesterol suggesting an interaction between CBD and cholesterol in lipid membranes (Perez et al., 2022).

Among more than 120 phytocannabinoids found in cannabis, to date, beside THC, some other cannabinoids such as cannabinol, (−)-trans-Δ^8^-tetrahydrocannabinol, (−)-trans-Δ^9^-tetrahydrocannabivarin, cannabidivarin, cannabigerol, cannabichromene, and the sesquiterpene (E)-β-caryophyllene have also been shown to bind and fully or partially activate cannabinoid receptors (Husni et al., 2014; Pertwee and Cascio, 2014; Zagzoog et al., 2020). Notably, majority of phytocannabinoids that do not interact with cannabinoids receptors and several phenolic terpenes found in the cannabis plant also act as allosteric modulators of various ion channels including several members of LGICs (Nurulain et al., 2015; Oz et al., 2015; Hoffmann et al., 2016; Lozon et al., 2016; Al Kury et al., 2018), suggesting that these compounds can also potentially contribute to allosteric effects of cannabis through cannabinoid receptor-independent mechanisms. Importantly, in addition to LGICs, cannabinoids act through cannabinoid-independent mechanisms to modulate the functions of TRP channels (Muller et al., 2018; Storozhuk and Zholos, 2018), PPARs (Iannotti and Vitale, 2021; Lago-Fernandez et al., 2021), 5-HT1A receptors (Russo et al., 2005; Rodrigues da Silva et al., 2020; Yano et al., 2020), and other receptors (Ibeas Bih et al., 2015; Morales et al., 2017; Turner et al., 2017).

Lipophilic features of cannabinoids markedly affect their pharmacokinetic properties as well. In biochemical studies, tissue AEA and 2-AG concentrations range between 10-214 pmol/g (9 nM–195 nM) and 1-21 nmol/g (1 µM–19 µM), respectively (Buczynski and Parsons, 2010). However, tissue endocannabinoid concentrations display significant spatiotemporal differences among brain regions and within the cell as well. Presumably, *de novo* synthesized endocannabinoids are preferentially incorporated into lipid membranes and give rise to spatially localized signaling within the cell. Similarly, phytocannabinoids, due to their high lipophilicity, are expected to attain membrane concentrations that are considerably higher than blood levels (Deiana et al., 2012). In an earlier study, it has been shown that perfusion of isolated rat hearts with buffer containing [^3^H]-CBD results in strong accumulation of radioactivity in the tissue (Smiley et al., 1976). Recently, chronic (28 days) administration of CBD (230 mg/kg) was shown to reach CBD concentrations of 2.7 µM and 3.3 µM in muscle and liver tissue, respectively (Child and Tallon, 2022). Therefore, the membrane concentrations of cannabinoids are likely to reach to pharmacologically relevant ranges to exert their effects on the functional properties of LGICs described in this review. However, the results of studies using concentrations of cannabinoids above 20-30 µM may have no pharmacological relevance.

## Conclusion

Cannabinoids have appeared as important modulators of diverse pathological and physiological processes and intensive research efforts have examined the efficacy of cannabinoid antagonist and agonist as therapeutic agents. Although cannabinoids primarily exert their cellular and organ system effects by interacting with CB1 and CB2 cannabinoid receptors, in recent years, it has been shown that not all effects of these agents are mediated by cannabinoid receptors. Several lines of evidence show that cannabinoids exert their effects by modulating activities of ion channels; transporters; enzymes, and other G-protein-coupled receptors in a cannabinoid receptor-independent manner. Among these diverse array of cellular macromolecules, ligand-gated ion channels constitute an important target that can effectively modulate neurotransmission in both central and peripheral nervous system.

## References

[B1] Ahrens J. Demir R. Leuwer M. de la Roche J. Krampfl K. Foadi N. (2009a). The nonpsychotropic cannabinoid cannabidiol modulates and directly activates alpha-1 and alpha-1-Beta glycine receptor function. Pharmacology 83 (4), 217–222. 10.1159/000201556 19204413

[B2] Ahrens J. Leuwer M. Demir R. Krampfl K. de la Roche J. Foadi N. (2009b). Positive allosteric modulatory effects of ajulemic acid at strychnine-sensitive glycine alpha1- and alpha1beta-receptors. Naunyn. Schmiedeb. Arch. Pharmacol. 379 (4), 371–378. 10.1007/s00210-008-0366-8 18985319

[B3] Aitta-Aho T. Maksimovic M. Dahl K. Sprengel R. Korpi E. R. (2019). Attenuation of novelty-induced hyperactivity of Gria1-/- mice by cannabidiol and hippocampal inhibitory chemogenetics. Front. Pharmacol. 10, 309. 10.3389/fphar.2019.00309 30984001 PMC6449460

[B4] Akinshola B. E. Taylor R. E. Ogunseitan A. B. Onaivi E. S. (1999). Anandamide inhibition of recombinant AMPA receptor subunits in Xenopus oocytes is increased by forskolin and 8-bromo-cyclic AMP. Naunyn. Schmiedeb. Arch. Pharmacol. 360 (3), 242–248. 10.1007/s002109900078 10543424

[B5] Al Kury L. T. Mahgoub M. Howarth F. C. Oz M. (2018). Natural negative allosteric modulators of 5-ht₃ receptors. Molecules 23 (12), E3186. 10.3390/molecules23123186 30513973 PMC6321066

[B6] Albuquerque E. X. Pereira E. F. Alkondon M. Rogers S. W. (2009). Mammalian nicotinic acetylcholine receptors: From structure to function. Physiol. Rev. 89 (1), 73–120. 10.1152/physrev.00015.2008 19126755 PMC2713585

[B7] Alexander S. P. Peters J. A. Kelly E. Marrion N. V. Faccenda E. Harding S. D. (2017). The concise guide to pharmacology 2017/18: Ligand-gated ion channels. Br. J. Pharmacol. 174 (1), S130–s159. 10.1111/bph.13879 29055038 PMC5650660

[B8] Alzaabi A. H. Howarth L. El Nebrisi E. Syed N. Susan Yang K. H. Howarth F. C. (2019). Capsaicin inhibits the function of α(7)-nicotinic acetylcholine receptors expressed in Xenopus oocytes and rat hippocampal neurons. Eur. J. Pharmacol. 857, 172411. 10.1016/j.ejphar.2019.172411 31152699

[B9] Ambrosi S. Ragni L. Ambrosini A. Paccamiccio L. Mariani P. Fiorini R. (2005). On the importance of anandamide structural features for its interactions with DPPC bilayers: Effects on PLA2 activity. J. Lipid Res. 46 (9), 1953–1961. 10.1194/jlr.M500121-JLR200 15961786

[B10] Anderson L. L. Absalom N. L. Abelev S. V. Low I. K. Doohan P. T. Martin L. J. (2019). Coadministered cannabidiol and clobazam: Preclinical evidence for both pharmacodynamic and pharmacokinetic interactions. Epilepsia 60 (11), 2224–2234. 10.1111/epi.16355 31625159 PMC6900043

[B11] Anderson L. L. Heblinski M. Absalom N. L. Hawkins N. A. Bowen M. T. Benson M. J. (2021). Cannabigerolic acid, a major biosynthetic precursor molecule in cannabis, exhibits divergent effects on seizures in mouse models of epilepsy. Br. J. Pharmacol. 178 (24), 4826–4841. 10.1111/bph.15661 34384142 PMC9292928

[B12] Bach D. Raz A. Goldman R. (1976). The interaction of hashish compounds with planar lipid bilayer membranes (BLM). Biochem. Pharmacol. 25 (11), 1241–1244. 10.1016/0006-2952(76)90084-8 938548

[B13] Bakas T. van Nieuwenhuijzen P. S. Devenish S. O. McGregor I. S. Arnold J. C. Chebib M. (2017). The direct actions of cannabidiol and 2-arachidonoyl glycerol at GABA(A) receptors. Pharmacol. Res. 119, 358–370. 10.1016/j.phrs.2017.02.022 28249817

[B14] Barann M. Molderings G. Brüss M. Bönisch H. Urban B. W. Göthert M. (2002). Direct inhibition by cannabinoids of human 5-ht3a receptors: Probable involvement of an allosteric modulatory site. Br. J. Pharmacol. 137 (5), 589–596. 10.1038/sj.bjp.0704829 12381672 PMC1573528

[B15] Baranowska U. Göthert M. Rudz R. Malinowska B. (2008). Methanandamide allosterically inhibits *in vivo* the function of peripheral nicotinic acetylcholine receptors containing the alpha 7-subunit. J. Pharmacol. Exp. Ther. 326 (3), 912–919. 10.1124/jpet.108.140863 18567834

[B16] Barnes N. M. Hales T. G. Lummis S. C. Peters J. A. (2009). The 5-HT3 receptor--the relationship between structure and function. Neuropharmacology 56 (1), 273–284. 10.1016/j.neuropharm.2008.08.003 18761359 PMC6485434

[B17] Barnett-Norris J. Lynch D. Reggio P. H. (2005). Lipids, lipid rafts and caveolae: Their importance for GPCR signaling and their centrality to the endocannabinoid system. Life Sci. 77 (14), 1625–1639. 10.1016/j.lfs.2005.05.040 15993425

[B18] Baskfield C. Y. Martin B. R. Wiley J. L. (2004). Differential effects of delta9-tetrahydrocannabinol and methanandamide in CB1 knockout and wild-type mice. J. Pharmacol. Exp. Ther. 309 (1), 86–91. 10.1124/jpet.103.055376 14718593

[B19] Baur R. Gertsch J. Sigel E. (2013a). Do N-arachidonyl-glycine (NA-glycine) and 2-arachidonoyl glycerol (2-AG) share mode of action and the binding site on the β2 subunit of GABAA receptors? PeerJ 1, e149. 10.7717/peerj.149 24058880 PMC3775635

[B20] Baur R. Gertsch J. Sigel E. (2012). The cannabinoid CB1 receptor antagonists rimonabant (SR141716) and AM251 directly potentiate GABA(A) receptors. Br. J. Pharmacol. 165 (8), 2479–2484. 10.1111/j.1476-5381.2011.01405.x 21470203 PMC3423234

[B21] Baur R. Kielar M. Richter L. Ernst M. Ecker G. F. Sigel E. (2013b). Molecular analysis of the site for 2-arachidonylglycerol (2-AG) on the β₂ subunit of GABA(A) receptors. J. Neurochem. 126 (1), 29–36. 10.1111/jnc.12270 23600744

[B22] Biala G. Kruk M. (2008). Cannabinoid receptor ligands suppress memory-related effects of nicotine in the elevated plus maze test in mice. Behav. Brain Res. 192 (2), 198–202. 10.1016/j.bbr.2008.04.004 18501975

[B23] Bloom A. S. Edgemond W. S. Moldvan J. C. (1997). Nonclassical and endogenous cannabinoids: Effects on the ordering of brain membranes. Neurochem. Res. 22 (5), 563–568. 10.1023/a:1022413901857 9131634

[B24] Borroni V. Barrantes F. J. (2021). Homomeric and heteromeric α7 nicotinic acetylcholine receptors in health and some central nervous system diseases. Membr. (Basel) 11 (9), 664. 10.3390/membranes11090664 PMC8465519 34564481

[B25] Bruggemann E. P. Melchior D. L. (1983). Alterations in the organization of phosphatidylcholine/cholesterol bilayers by tetrahydrocannabinol. J. Biol. Chem. 258 (13), 8298–8303. 10.1016/s0021-9258(20)82064-x 6305981

[B26] Buczynski M. W. Parsons L. H. (2010). Quantification of brain endocannabinoid levels: Methods, interpretations and pitfalls. Br. J. Pharmacol. 160 (3), 423–442. 10.1111/j.1476-5381.2010.00787.x 20590555 PMC2931546

[B27] Burgos C. F. Yévenes G. E. Aguayo L. G. (2016). Structure and pharmacologic modulation of inhibitory Glycine receptors. Mol. Pharmacol. 90 (3), 318–325. 10.1124/mol.116.105726 27401877 PMC4998662

[B28] Butt C. Alptekin A. Shippenberg T. Oz M. (2008). Endogenous cannabinoid anandamide inhibits nicotinic acetylcholine receptor function in mouse thalamic synaptosomes. J. Neurochem. 105 (4), 1235–1243. 10.1111/j.1471-4159.2008.05225.x 18194436

[B29] Chen J. Lee C. T. Errico S. Deng X. Cadet J. L. Freed W. J. (2005). Protective effects of Delta(9)-tetrahydrocannabinol against N-methyl-d-aspartate-induced AF5 cell death. Brain Res. Mol. Brain Res. 134 (2), 215–225. 10.1016/j.molbrainres.2004.10.044 15836919 PMC1824211

[B30] Child R. B. Tallon M. J. (2022). Cannabidiol (CBD) dosing: Plasma pharmacokinetics and effects on accumulation in skeletal muscle, liver and adipose tissue. Nutrients 14 (10), 2101. 10.3390/nu14102101 35631242 PMC9146469

[B31] Cifelli P. Ruffolo G. De Felice E. Alfano V. van Vliet E. A. Aronica E. (2020). Phytocannabinoids in neurological diseases: Could they restore a physiological GABAergic transmission? Int. J. Mol. Sci. 21 (3), E723. 10.3390/ijms21030723 31979108 PMC7038116

[B32] Cristino L. Bisogno T. Di Marzo V. (2020). Cannabinoids and the expanded endocannabinoid system in neurological disorders. Nat. Rev. Neurol. 16 (1), 9–29. 10.1038/s41582-019-0284-z 31831863

[B33] Dainese E. Sabatucci A. Angelucci C. B. Barsacchi D. Chiarini M. Maccarrone M. (2012). Impact of embedded endocannabinoids and their oxygenation by lipoxygenase on membrane properties. ACS Chem. Neurosci. 3 (5), 386–392. 10.1021/cn300016c 22860207 PMC3386857

[B34] Deiana S. Watanabe A. Yamasaki Y. Amada N. Arthur M. Fleming S. (2012). Plasma and brain pharmacokinetic profile of cannabidiol (CBD), cannabidivarine (CBDV), Δ⁹-tetrahydrocannabivarin (THCV) and cannabigerol (CBG) in rats and mice following oral and intraperitoneal administration and CBD action on obsessive-compulsive behaviour. Psychopharmacol. Berl. 219 (3), 859–873. 10.1007/s00213-011-2415-0 21796370

[B35] Demir R. Leuwer M. de la Roche J. Krampfl K. Foadi N. Karst M. (2009). Modulation of glycine receptor function by the synthetic cannabinoid HU210. Pharmacology 83 (5), 270–274. 10.1159/000209291 19307742

[B36] Devane W. A. Hanus L. Breuer A. Pertwee R. G. Stevenson L. A. Griffin G. (1992). Isolation and structure of a brain constituent that binds to the cannabinoid receptor. Science 258 (5090), 1946–1949. 10.1126/science.1470919 1470919

[B37] Di Marzo V. Breivogel C. S. Tao Q. Bridgen D. T. Razdan R. K. Zimmer A. M. (2000). Levels, metabolism, and pharmacological activity of anandamide in CB(1) cannabinoid receptor knockout mice: Evidence for non-CB(1), non-CB(2) receptor-mediated actions of anandamide in mouse brain. J. Neurochem. 75 (6), 2434–2444. 10.1046/j.1471-4159.2000.0752434.x 11080195

[B38] Di Marzo V. Fontana A. Cadas H. Schinelli S. Cimino G. Schwartz J. C. (1994). Formation and inactivation of endogenous cannabinoid anandamide in central neurons. Nature 372 (6507), 686–691. 10.1038/372686a0 7990962

[B39] Di Pasquale E. Chahinian H. Sanchez P. Fantini J. (2009). The insertion and transport of anandamide in synthetic lipid membranes are both cholesterol-dependent. PLoS One 4 (3), e4989. 10.1371/journal.pone.0004989 19330032 PMC2658885

[B40] Dineley K. T. Pandya A. A. Yakel J. L. (2015). Nicotinic ACh receptors as therapeutic targets in CNS disorders. Trends Pharmacol. Sci. 36 (2), 96–108. 10.1016/j.tips.2014.12.002 25639674 PMC4324614

[B41] ElSohly M. A. Radwan M. M. Gul W. Chandra S. Galal A. (2017). Phytochemistry of cannabis sativa L. Prog. Chem. Org. Nat. Prod. 103, 1–36. 10.1007/978-3-319-45541-9_1 28120229

[B42] Faerber L. Drechsler S. Ladenburger S. Gschaidmeier H. Fischer W. (2007). The neuronal 5-HT3 receptor network after 20 years of research--evolving concepts in management of pain and inflammation. Eur. J. Pharmacol. 560 (1), 1–8. 10.1016/j.ejphar.2007.01.028 17316606

[B43] Fan P. (1995). Cannabinoid agonists inhibit the activation of 5-HT3 receptors in rat nodose ganglion neurons. J. Neurophysiol. 73 (2), 907–910. 10.1152/jn.1995.73.2.907 7760148

[B44] Feigenbaum J. J. Bergmann F. Richmond S. A. Mechoulam R. Nadler V. Kloog Y. (1989). Nonpsychotropic cannabinoid acts as a functional N-methyl-D-aspartate receptor blocker. Proc. Natl. Acad. Sci. U. S. A. 86 (23), 9584–9587. 10.1073/pnas.86.23.9584 2556719 PMC298542

[B45] Foadi N. Leuwer M. Demir R. Dengler R. Buchholz V. de la Roche J. (2010). Lack of positive allosteric modulation of mutated alpha(1)S267I glycine receptors by cannabinoids. Naunyn. Schmiedeb. Arch. Pharmacol. 381 (5), 477–482. 10.1007/s00210-010-0506-9 20339834

[B46] Fowler C. J. Doherty P. Alexander S. P. H. (2017). Endocannabinoid turnover. Adv. Pharmacol. 80, 31–66. 10.1016/bs.apha.2017.03.006 28826539

[B47] Ghit A. Assal D. Al-Shami A. S. Hussein D. E. E. (2021). GABA(A) receptors: Structure, function, pharmacology, and related disorders. J. Genet. Eng. Biotechnol. 19 (1), 123. 10.1186/s43141-021-00224-0 34417930 PMC8380214

[B48] Ghovanloo M. R. Choudhury K. Bandaru T. S. Fouda M. A. Rayani K. Rusinova R. (2021). Cannabidiol inhibits the skeletal muscle Nav1.4 by blocking its pore and by altering membrane elasticity. J. Gen. Physiol. 153 (5), e202012701. 10.1085/jgp.202012701 33836525 PMC8042605

[B49] Ghovanloo M. R. Estacion M. Higerd-Rusli G. P. Zhao P. Dib-Hajj S. Waxman S. G. (2022). Inhibition of sodium conductance by cannabigerol contributes to a reduction of dorsal root ganglion neuron excitability. Br. J. Pharmacol. 179 (15), 4010–4030. 10.1111/bph.15833 35297036 PMC13012276

[B50] Ghovanloo M. R. Ruben P. C. (2021). Cannabidiol and sodium channel pharmacology: General overview, mechanism, and clinical implications. Neuroscientist. 28, 318–334. 10.1177/10738584211017009 34027742 PMC9344566

[B51] Godlewski G. Göthert M. Malinowska B. (2003). Cannabinoid receptor-independent inhibition by cannabinoid agonists of the peripheral 5-HT3 receptor-mediated von Bezold-Jarisch reflex. Br. J. Pharmacol. 138 (5), 767–774. 10.1038/sj.bjp.0705114 12642377 PMC1573725

[B52] Golovko T. Min R. Lozovaya N. Falconer C. Yatsenko N. Tsintsadze T. (2015). Control of inhibition by the direct action of cannabinoids on GABAA receptors. Cereb. Cortex 25 (9), 2440–2455. 10.1093/cercor/bhu045 24646614

[B53] Hampson A. J. Bornheim L. M. Scanziani M. Yost C. S. Gray A. T. Hansen B. M. (1998). Dual effects of anandamide on NMDA receptor-mediated responses and neurotransmission. J. Neurochem. 70 (2), 671–676. 10.1046/j.1471-4159.1998.70020671.x 9453561

[B54] Hansen K. B. Wollmuth L. P. Bowie D. Furukawa H. Menniti F. S. Sobolevsky A. I. (2021). Structure, function, and pharmacology of glutamate receptor ion channels. Pharmacol. Rev. 73 (4), 1469–1658. 10.1124/pharmrev.120.000131 PMC8626789 34753794

[B55] Hejazi N. Zhou C. Oz M. Sun H. Ye J. H. Zhang L. (2006). Delta9-tetrahydrocannabinol and endogenous cannabinoid anandamide directly potentiate the function of glycine receptors. Mol. Pharmacol. 69 (3), 991–997. 10.1124/mol.105.019174 16332990

[B56] Hillard C. J. Harris R. A. Bloom A. S. (1985). Effects of the cannabinoids on physical properties of brain membranes and phospholipid vesicles: Fluorescence studies. J. Pharmacol. Exp. Ther. 232 (3), 579–588. 2983062

[B57] Hoffman P. W. Ravindran A. Huganir R. L. (1994). Role of phosphorylation in desensitization of acetylcholine receptors expressed in Xenopus oocytes. J. Neurosci. 14 (7), 4185–4195. 10.1523/jneurosci.14-07-04185.1994 8027770 PMC6577029

[B58] Hoffmann K. M. Herbrechter R. Ziemba P. M. Lepke P. Beltrán L. Hatt H. (2016). Kampo medicine: Evaluation of the pharmacological activity of 121 herbal drugs on GABAA and 5-ht3a receptors. Front. Pharmacol. 7, 219. 10.3389/fphar.2016.00219 27524967 PMC4965468

[B59] Howlett A. C. Thomas B. F. Huffman J. W. (2021). The spicy story of cannabimimetic indoles. Molecules 26 (20), 6190. 10.3390/molecules26206190 34684770 PMC8538531

[B60] Huffman J. W. Padgett L. W. (2005). Recent developments in the medicinal chemistry of cannabimimetic indoles, pyrroles and indenes. Curr. Med. Chem. 12 (12), 1395–1411. 10.2174/0929867054020864 15974991

[B61] Husni A. S. McCurdy C. R. Radwan M. M. Ahmed S. A. Slade D. Ross S. A. (2014). Evaluation of phytocannabinoids from high potency cannabis sativa using *in vitro* bioassays to determine structure-activity relationships for cannabinoid receptor 1 and cannabinoid receptor 2. Med. Chem. Res. 23 (9), 4295–4300. 10.1007/s00044-014-0972-6 25419092 PMC4235762

[B62] Iannotti F. A. Vitale R. M. (2021). The endocannabinoid system and PPARs: Focus on their signalling crosstalk, action and transcriptional regulation. Cells 10 (3), 586. 10.3390/cells10030586 33799988 PMC8001692

[B63] Iatsenko N. M. Tsintsadze T. Lozova N. O. (2007). The synthetic cannabinoid analog WIN 55, 212-2 potentiates the amplitudes of glycine-activated currents. Fiziol. Zh. 53 (3), 31–37. 17725041

[B64] Ibeas Bih C. Chen T. Nunn A. V. Bazelot M. Dallas M. Whalley B. J. (2015). Molecular targets of cannabidiol in neurological disorders. Neurotherapeutics 12 (4), 699–730. 10.1007/s13311-015-0377-3 26264914 PMC4604182

[B65] Isaev D. Shabbir W. Dinc E. Y. Lorke D. E. Petroianu G. Oz M. (2022). Cannabidiol inhibits multiple ion channels in rabbit ventricular cardiomyocytes. Front. Pharmacol. 13, 821758. 10.3389/fphar.2022.821758 35185573 PMC8850628

[B66] Izzo A. A. Borrelli F. Capasso R. Di Marzo V. Mechoulam R. (2009). Non-psychotropic plant cannabinoids: New therapeutic opportunities from an ancient herb. Trends Pharmacol. Sci. 30 (10), 515–527. 10.1016/j.tips.2009.07.006 19729208

[B67] Jackson S. N. Singhal S. K. Woods A. S. Morales M. Shippenberg T. Zhang L. (2008). Volatile anesthetics and endogenous cannabinoid anandamide have additive and independent inhibitory effects on alpha(7)-nicotinic acetylcholine receptor-mediated responses in Xenopus oocytes. Eur. J. Pharmacol. 582 (1-3), 42–51. 10.1016/j.ejphar.2007.12.023 18242598 PMC2346594

[B68] James T. R. Richards A. A. Lowe D. A. Reid W. A. Watson C. T. Pepple D. J. (2022). The *in vitro* effect of delta-9-tetrahydrocannabinol and cannabidiol on whole blood viscosity, elasticity and membrane integrity. J. Cannabis Res. 4 (1), 15. 10.1186/s42238-022-00126-z 35382895 PMC8981745

[B69] Järbe T. U. DiPatrizio N. V. Li C. Makriyannis A. (2003a). The cannabinoid receptor antagonist SR-141716 does not readily antagonize open-field effects induced by the cannabinoid receptor agonist (R)-methanandamide in rats. Pharmacol. Biochem. Behav. 75 (4), 809–821. 10.1016/s0091-3057(03)00168-0 12957223

[B70] Järbe T. U. Lamb R. J. Liu Q. Makriyannis A. (2003b). (R)-Methanandamide and delta9-tetrahydrocannabinol-induced operant rate decreases in rats are not readily antagonized by SR-141716A. Eur. J. Pharmacol. 466 (1-2), 121–127. 10.1016/s0014-2999(03)01491-2 12679148

[B71] Johnson D. E. Heald S. L. Dally R. D. Janis R. A. (1993). Isolation, identification and synthesis of an endogenous arachidonic amide that inhibits calcium channel antagonist 1, 4-dihydropyridine binding. Prostagl. Leukot. Essent. Fat. Acids 48 (6), 429–437. 10.1016/0952-3278(93)90048-2 8341720

[B72] Koe B. K. Milne G. M. Weissman A. Johnson M. R. Melvin L. S. (1985). Enhancement of brain [3H]flunitrazepam binding and analgesic activity of synthetic cannabimimetics. Eur. J. Pharmacol. 109 (2), 201–212. 10.1016/0014-2999(85)90421-2 2986995

[B73] Koe B. K. Weissman A. (1981). Facilitation of benzodiazepine binding by levonantradol. J. Clin. Pharmacol. 21 (S1), 397S-405S–405s. 10.1002/j.1552-4604.1981.tb02619.x 6117577

[B74] Kossakowski R. Schlicker E. Toczek M. Weresa J. Malinowska B. (2019). Cannabidiol affects the bezold-jarisch reflex via TRPV1 and 5-HT(3) receptors and has peripheral sympathomimetic effects in spontaneously hypertensive and normotensive rats. Front. Pharmacol. 10, 500. 10.3389/fphar.2019.00500 31178718 PMC6538767

[B75] Lagalwar S. Bordayo E. Z. Hoffmann K. L. Fawcett J. R. Frey W. H. 2nd (1999). Anandamides inhibit binding to the muscarinic acetylcholine receptor. J. Mol. Neurosci. 13 (1-2), 55–61. 10.1385/jmn:13:1-2:55 10691292

[B76] Lago-Fernandez A. Zarzo-Arias S. Jagerovic N. Morales P. (2021). Relevance of peroxisome proliferator activated receptors in multitarget paradigm associated with the endocannabinoid system. Int. J. Mol. Sci. 22 (3), 1001. 10.3390/ijms22031001 33498245 PMC7863932

[B77] Le Boisselier R. Alexandre J. Lelong-Boulouard V. Debruyne D. (2017). Focus on cannabinoids and synthetic cannabinoids. Clin. Pharmacol. Ther. 101 (2), 220–229. 10.1002/cpt.563 27861784

[B78] Lorke D. E. Petroianu G. Oz M. (2016). “α7-Nicotinic acetylcholine receptors and β-amyloid peptides in alzheimer’s disease,” in Nicotinic acetylcholine receptor technologies. Neuromethods. Editor Li M. (New York, NY, USA: Springer Science-Humana Press), 171–206.

[B79] Lozon Y. Sultan A. Lansdell S. J. Prytkova T. Sadek B. Yang K. H. (2016). Inhibition of human α7 nicotinic acetylcholine receptors by cyclic monoterpene carveol. Eur. J. Pharmacol. 776, 44–51. 10.1016/j.ejphar.2016.02.004 26849939

[B80] Lozovaya N. Mukhtarov M. Tsintsadze T. Ledent C. Burnashev N. Bregestovski P. (2011). Frequency-dependent cannabinoid receptor-independent modulation of Glycine receptors by endocannabinoid 2-AG. Front. Mol. Neurosci. 4, 13. 10.3389/fnmol.2011.00013 21847369 PMC3147161

[B81] Lozovaya N. Yatsenko N. Beketov A. Tsintsadze T. Burnashev N. (2005). Glycine receptors in CNS neurons as a target for nonretrograde action of cannabinoids. J. Neurosci. 25 (33), 7499–7506. 10.1523/jneurosci.0977-05.2005 16107637 PMC6725404

[B82] Lu J. Fan S. Zou G. Hou Y. Pan T. Guo W. (2018). Involvement of glycine receptor α1 subunits in cannabinoid-induced analgesia. Neuropharmacology 133, 224–232. 10.1016/j.neuropharm.2018.01.041 29407767

[B83] Lu Y. Liu C. Yang H. (2011). Inhibitory effects of synthetic cannabinoid WIN55, 212-2 on nicotine-activated currents in rat trigeminal ganglion neurons. Neural Regen. Res. 6 (8), 610–616.

[B84] Lummis S. C. (2012). 5-HT(3) receptors. J. Biol. Chem. 287 (48), 40239–40245. 10.1074/jbc.R112.406496 23038271 PMC3504740

[B85] Lundbaek J. A. Birn P. Tape S. E. Toombes G. E. Søgaard R. Koeppe R. E. 2nd (2005). Capsaicin regulates voltage-dependent sodium channels by altering lipid bilayer elasticity. Mol. Pharmacol. 68 (3), 680–689. 10.1124/mol.105.013573 15967874

[B86] Lynch J. W. (2009). Native glycine receptor subtypes and their physiological roles. Neuropharmacology 56 (1), 303–309. 10.1016/j.neuropharm.2008.07.034 18721822

[B87] Lynch J. W. Zhang Y. Talwar S. Estrada-Mondragon A. (2017). Glycine receptor drug discovery. Adv. Pharmacol. 79, 225–253. 10.1016/bs.apha.2017.01.003 28528670

[B88] Machu T. K. (2011). Therapeutics of 5-HT3 receptor antagonists: Current uses and future directions. Pharmacol. Ther. 130 (3), 338–347. 10.1016/j.pharmthera.2011.02.003 21356241 PMC3103470

[B89] Mahgoub M. Keun-Hang S. Y. Sydorenko V. Ashoor A. Kabbani N. Al Kury L. (2013). Effects of cannabidiol on the function of α7-nicotinic acetylcholine receptors. Eur. J. Pharmacol. 720 (1-3), 310–319. 10.1016/j.ejphar.2013.10.011 24140434

[B90] Makriyannis A. Tian X. Guo J. (2005). How lipophilic cannabinergic ligands reach their receptor sites. Prostagl. Other Lipid Mediat. 77 (1-4), 210–218. 10.1016/j.prostaglandins.2004.01.010 16099405

[B91] Makriyannis A. Yang D. P. Griffin R. G. Das Gupta S. K. (1990). The perturbation of model membranes by (-)-delta 9-tetrahydrocannabinol. Studies using solid-state 2H- and 13C-NMR. Biochim. Biophys. Acta 1028 (1), 31–42. 10.1016/0005-2736(90)90262-m 2169880

[B92] Malinowska B. Zakrzeska A. Kurz C. M. Göthert M. Kwolek G. Wielgat P. (2010). Involvement of central beta2-adrenergic, NMDA and thromboxane A2 receptors in the pressor effect of anandamide in rats. Naunyn. Schmiedeb. Arch. Pharmacol. 381 (4), 349–360. 10.1007/s00210-010-0497-6 20198363

[B93] Martin L. J. Banister S. D. Bowen M. T. (2021). Understanding the complex pharmacology of cannabidiol: Mounting evidence suggests a common binding site with cholesterol. Pharmacol. Res. 166, 105508. 10.1016/j.phrs.2021.105508 33610721

[B94] Mavromoustakos T. Papahatjis D. Laggner P. (2001). Differential membrane fluidization by active and inactive cannabinoid analogues. Biochim. Biophys. Acta 1512 (2), 183–190. 10.1016/s0005-2736(01)00315-7 11406095

[B95] Mechoulam R. Ben-Shabat S. Hanus L. Ligumsky M. Kaminski N. E. Schatz A. R. (1995). Identification of an endogenous 2-monoglyceride, present in canine gut, that binds to cannabinoid receptors. Biochem. Pharmacol. 50 (1), 83–90. 10.1016/0006-2952(95)00109-d 7605349

[B96] Medeiros D. Silva-Gonçalves L. C. da Silva A. M. Dos Santos Cabrera M. P. Arcisio-Miranda M. (2017). Membrane-mediated action of the endocannabinoid anandamide on membrane proteins: Implications for understanding the receptor-independent mechanism. Sci. Rep. 7, 41362. 10.1038/srep41362 28128290 PMC5269673

[B97] Merritt L. L. Martin B. R. Walters C. Lichtman A. H. Damaj M. I. (2008). The endogenous cannabinoid system modulates nicotine reward and dependence. J. Pharmacol. Exp. Ther. 326 (2), 483–492. 10.1124/jpet.108.138321 18451315 PMC2746999

[B98] Mirlohi S. Bladen C. Santiago M. J. Arnold J. C. McGregor I. Connor M. (2022). Inhibition of human recombinant T-type calcium channels by phytocannabinoids *in vitro* . Br. J. Pharmacol. 179 (15), 4031–4043. 10.1111/bph.15842 35342937

[B99] Mizrachi T. Vaknin-Dembinsky A. Brenner T. Treinin M. (2021). Neuroinflammation modulation via α7 nicotinic acetylcholine receptor and its chaperone, RIC-3. Molecules 26 (20), 6139. 10.3390/molecules26206139 34684720 PMC8539643

[B100] Mokler D. J. Nelson B. D. Harris L. S. Rosecrans J. A. (1986). The role of benzodiazepine receptors in the discriminative stimulus properties of delta-9-tetrahydrocannabinol. Life Sci. 38 (17), 1581–1589. 10.1016/0024-3205(86)90497-2 3010019

[B101] Morales M. Bäckman C. (2002). Coexistence of serotonin 3 (5-HT3) and CB1 cannabinoid receptors in interneurons of hippocampus and dentate gyrus. Hippocampus 12 (6), 756–764. 10.1002/hipo.10025 12542227

[B102] Morales M. Wang S. D. Diaz-Ruiz O. Jho D. H. (2004). Cannabinoid CB1 receptor and serotonin 3 receptor subunit A (5-HT3A) are co-expressed in GABA neurons in the rat telencephalon. J. Comp. Neurol. 468 (2), 205–216. 10.1002/cne.10968 14648680

[B103] Morales M. Wang S. D. (2002). Differential composition of 5-hydroxytryptamine3 receptors synthesized in the rat CNS and peripheral nervous system. J. Neurosci. 22 (15), 6732–6741. 12151552 10.1523/JNEUROSCI.22-15-06732.2002 PMC6758137

[B104] Morales P. Hurst D. P. Reggio P. H. (2017). Molecular targets of the phytocannabinoids: A complex picture. Prog. Chem. Org. Nat. Prod. 103, 103–131. 10.1007/978-3-319-45541-9_4 28120232 PMC5345356

[B105] Morano A. Cifelli P. Nencini P. Antonilli L. Fattouch J. Ruffolo G. (2016). Cannabis in epilepsy: From clinical practice to basic research focusing on the possible role of cannabidivarin. Epilepsia Open 1 (3-4), 145–151. 10.1002/epi4.12015 29588939 PMC5719834

[B106] Muller C. Morales P. Reggio P. H. (2018). Cannabinoid ligands targeting TRP channels. Front. Mol. Neurosci. 11, 487. 10.3389/fnmol.2018.00487 30697147 PMC6340993

[B107] Nebrisi E. E. Prytkova T. Lorke D. E. Howarth L. Alzaabi A. H. Yang K. S. (2020). Capsaicin is a negative allosteric modulator of the 5-HT(3) receptor. Front. Pharmacol. 11, 1274. 10.3389/fphar.2020.01274 32982728 PMC7490547

[B108] Nishizaki T. Sumikawa K. (1998). Effects of PKC and PKA phosphorylation on desensitization of nicotinic acetylcholine receptors. Brain Res. 812 (1-2), 242–245. 10.1016/s0006-8993(98)00836-1 9813350

[B109] Nurulain S. Prytkova T. Sultan A. M. Ievglevskyi O. Lorke D. Yang K. H. (2015). Inhibitory actions of bisabolol on α7-nicotinic acetylcholine receptors. Neuroscience 306, 91–99. 10.1016/j.neuroscience.2015.08.019 26283025

[B110] Olsen R. W. (2018). GABA(A) receptor: Positive and negative allosteric modulators. Neuropharmacology 136, 10–22. 10.1016/j.neuropharm.2018.01.036 29407219 PMC6027637

[B111] Olsen R. W. Sieghart W. (2008). International union of pharmacology. LXX. Subtypes of gamma-aminobutyric acid(A) receptors: Classification on the basis of subunit composition, pharmacology, and function. Update. Pharmacol. Rev. 60 (3), 243–260. 10.1124/pr.108.00505 18790874 PMC2847512

[B112] Ortiz Y. T. McMahon L. R. Wilkerson J. L. (2022). Medicinal cannabis and central nervous system disorders. Front. Pharmacol. 13, 881810. 10.3389/fphar.2022.881810 35529444 PMC9070567

[B113] Oz M. Al Kury L. Keun-Hang S. Y. Mahgoub M. Galadari S. (2014). Cellular approaches to the interaction between cannabinoid receptor ligands and nicotinic acetylcholine receptors. Eur. J. Pharmacol. 731, 100–105. 10.1016/j.ejphar.2014.03.010 24642359

[B114] Oz M. Brauneis U. Zhang L. Weight F. F. (1995). Inhibition by the endogenous cannabinoid anandamide, of 5-HT3 receptor-mediated ion current in Xenopus oocytes. Proc. 3rd Eur. Symposium Drug Addict. AIDS 1, 64.

[B115] Oz M. Jackson S. N. Woods A. S. Morales M. Zhang L. (2005). Additive effects of endogenous cannabinoid anandamide and ethanol on alpha7-nicotinic acetylcholine receptor-mediated responses in Xenopus Oocytes. J. Pharmacol. Exp. Ther. 313 (3), 1272–1280. 10.1124/jpet.104.081315 15687372

[B116] Oz M. Lorke D. E. Yang K. H. Petroianu G. (2013). On the interaction of β-amyloid peptides and α7-nicotinic acetylcholine receptors in Alzheimer's disease. Curr. Alzheimer Res. 10 (6), 618–630. 10.2174/15672050113109990132 23627750

[B117] Oz M. Lozon Y. Sultan A. Yang K. H. Galadari S. (2015). Effects of monoterpenes on ion channels of excitable cells. Pharmacol. Ther. 152, 83–97. 10.1016/j.pharmthera.2015.05.006 25956464

[B118] Oz M. Petroianu G. Lorke D. E. (2016). “α7-Nicotinic acetylcholine receptors: New therapeutic avenues in alzheimer’s disease,” in Nicotinic acetylcholine receptor technologies. Neuromethods. Editor Li M. (New York, NY: USA Springer Science-Humana Press), 149–170.

[B119] Oz M. Ravindran A. Diaz-Ruiz O. Zhang L. Morales M. (2003). The endogenous cannabinoid anandamide inhibits alpha7 nicotinic acetylcholine receptor-mediated responses in Xenopus oocytes. J. Pharmacol. Exp. Ther. 306 (3), 1003–1010. 10.1124/jpet.103.049981 12766252

[B120] Oz M. (2006). Receptor-independent actions of cannabinoids on cell membranes: Focus on endocannabinoids. Pharmacol. Ther. 111 (1), 114–144. 10.1016/j.pharmthera.2005.09.009 16584786

[B121] Oz M. Tchugunova Y. B. Dunn S. M. (2000). Endogenous cannabinoid anandamide directly inhibits voltage-dependent Ca(2+) fluxes in rabbit T-tubule membranes. Eur. J. Pharmacol. 404 (1-2), 13–20. 10.1016/s0014-2999(00)00396-4 10980258

[B122] Oz M. Tchugunova Y. Dinc M. (2004a). Differential effects of endogenous and synthetic cannabinoids on voltage-dependent calcium fluxes in rabbit T-tubule membranes: Comparison with fatty acids. Eur. J. Pharmacol. 502 (1-2), 47–58. 10.1016/j.ejphar.2004.08.052 15464089

[B123] Oz M. Zhang L. Morales M. (2002a). Endogenous cannabinoid, anandamide, acts as a noncompetitive inhibitor on 5-HT3 receptor-mediated responses in Xenopus oocytes. Synapse 46 (3), 150–156. 10.1002/syn.10121 12325042

[B124] Oz M. Zhang L. Ravindran A. Morales M. Lupica C. R. (2004b). Differential effects of endogenous and synthetic cannabinoids on alpha7-nicotinic acetylcholine receptor-mediated responses in Xenopus Oocytes. J. Pharmacol. Exp. Ther. 310 (3), 1152–1160. 10.1124/jpet.104.067751 15102930

[B125] Oz M. Zhang L. Spivak C. E. (2002b). Direct noncompetitive inhibition of 5-HT(3) receptor-mediated responses by forskolin and steroids. Arch. Biochem. Biophys. 404 (2), 293–301. 10.1016/s0003-9861(02)00279-5 12147268

[B126] Pagano C. Navarra G. Coppola L. Avilia G. Bifulco M. Laezza C. (2022). Cannabinoids: Therapeutic use in clinical practice. Int. J. Mol. Sci. 23 (6), 3344. 10.3390/ijms23063344 35328765 PMC8952215

[B127] Papke R. L. Horenstein N. A. (2021). Therapeutic targeting of α7 nicotinic acetylcholine receptors. Pharmacol. Rev. 73 (3), 1118–1149. 10.1124/pharmrev.120.000097 34301823 PMC8318519

[B128] Perez E. Ceja-Vega J. Krmic M. Gamez Hernandez A. Gudyka J. Porteus R. (2022). Differential interaction of cannabidiol with biomembranes dependent on cholesterol concentration. ACS Chem. Neurosci. 13 (7), 1046–1054. 10.1021/acschemneuro.2c00040 35298887

[B129] Pertwee R. G. Cascio M. G. (2014). “Known pharmacological actions of delta-9-tetrahydrocannabinol and of four other chemical constituents of cannabis that activate cannabinoid receptors,” in Handbook of cannabis. Editor Pertwee R. G. (Oxford, UK: Oxford University Press), 115–136.

[B130] Pertwee R. G. Howlett A. C. Abood M. E. Alexander S. P. Di Marzo V. Elphick M. R. (2010). International union of basic and clinical pharmacology. LXXIX. Cannabinoid receptors and their ligands: Beyond CB₁ and CB₂. Pharmacol. Rev. 62 (4), 588–631. 10.1124/pr.110.003004 21079038 PMC2993256

[B131] Plested A. J. (2016). Structural mechanisms of activation and desensitization in neurotransmitter-gated ion channels. Nat. Struct. Mol. Biol. 23 (6), 494–502. 10.1038/nsmb.3214 27273633

[B132] Przegaliński E. Göthert M. Frankowska M. Filip M. (2005). WIN 55, 212-2-induced reduction of cocaine hyperlocomotion: Possible inhibition of 5-HT(3) receptor function. Eur. J. Pharmacol. 517 (1-2), 68–73. 10.1016/j.ejphar.2005.05.014 15961074

[B133] Rácz I. Bilkei-Gorzo A. Markert A. Stamer F. Göthert M. Zimmer A. (2008). Anandamide effects on 5-HT(3) receptors *in vivo* . Eur. J. Pharmacol. 596 (1-3), 98–101. 10.1016/j.ejphar.2008.08.012 18775693

[B134] Reggio P. H. Traore H. (2000). Conformational requirements for endocannabinoid interaction with the cannabinoid receptors, the anandamide transporter and fatty acid amidohydrolase. Chem. Phys. Lipids 108 (1-2), 15–35. 10.1016/s0009-3084(00)00185-7 11106780

[B135] Robinson L. Goonawardena A. V. Pertwee R. Hampson R. E. Platt B. Riedel G. (2010). WIN55, 212-2 induced deficits in spatial learning are mediated by cholinergic hypofunction. Behav. Brain Res. 208 (2), 584–592. 10.1016/j.bbr.2010.01.004 20079375 PMC3151156

[B136] Rodrigues da Silva N. Gomes F. V. Sonego A. B. Silva N. R. D. Guimarães F. S. (2020). Cannabidiol attenuates behavioral changes in a rodent model of schizophrenia through 5-HT1A, but not CB1 and CB2 receptors. Pharmacol. Res. 156, 104749. 10.1016/j.phrs.2020.104749 32151683

[B137] Ruffolo G. Cifelli P. Roseti C. Thom M. van Vliet E. A. Limatola C. (2018). A novel GABAergic dysfunction in human Dravet syndrome. Epilepsia 59 (11), 2106–2117. 10.1111/epi.14574 30306542

[B138] Russo E. B. Burnett A. Hall B. Parker K. K. (2005). Agonistic properties of cannabidiol at 5-HT1a receptors. Neurochem. Res. 30 (8), 1037–1043. 10.1007/s11064-005-6978-1 16258853

[B139] Sanger G. J. Andrews P. L. (2006). Treatment of nausea and vomiting: Gaps in our knowledge. Auton. Neurosci. 129 (1-2), 3–16. 10.1016/j.autneu.2006.07.009 16934536

[B140] Senn L. Cannazza G. Biagini G. (2020). Receptors and channels possibly mediating the effects of phytocannabinoids on seizures and epilepsy. Pharm. (Basel) 13 (8), E174. 10.3390/ph13080174 PMC7463541 32751761

[B141] Shi B. Yang R. Wang X. Liu H. Zou L. Hu X. (2012). Inhibition of 5-HT(3) receptors-activated currents by cannabinoids in rat trigeminal ganglion neurons. J. Huazhong Univ. Sci. Technol. Med. Sci. 32 (2), 265–271. 10.1007/s11596-012-0047-1 22528232

[B142] Siara J. Ruppersberg J. P. Rüdel R. (1990). Human nicotinic acetylcholine receptor: The influence of second messengers on activation and desensitization. Pflugers Arch. 415 (6), 701–706. 10.1007/bf02584008 2159619

[B143] Sigel E. Baur R. Rácz I. Marazzi J. Smart T. G. Zimmer A. (2011). The major central endocannabinoid directly acts at GABA(A) receptors. Proc. Natl. Acad. Sci. U. S. A. 108 (44), 18150–18155. 10.1073/pnas.1113444108 22025726 PMC3207709

[B144] Sigel E. Steinmann M. E. (2012). Structure, function, and modulation of GABA(A) receptors. J. Biol. Chem. 287 (48), 40224–40231. 10.1074/jbc.R112.386664 23038269 PMC3504738

[B145] Smiley K. A. Karler R. Turkanis S. A. (1976). Effects of cannabinoids on the perfused rat heart. Res. Commun. Chem. Pathol. Pharmacol. 14 (4), 659–675. 959665

[B146] Smith L. C. Tieu L. Suhandynata R. T. Boomhower B. Hoffman M. Sepulveda Y. (2021). Cannabidiol reduces withdrawal symptoms in nicotine-dependent rats. Psychopharmacol. Berl. 238 (8), 2201–2211. 10.1007/s00213-021-05845-4 PMC8295227 33909102

[B147] Soderstrom K. Soliman E. Van Dross R. (2017). Cannabinoids modulate neuronal activity and cancer by CB1 and CB2 receptor-independent mechanisms. Front. Pharmacol. 8, 720. 10.3389/fphar.2017.00720 29066974 PMC5641363

[B148] Spivak C. E. Lupica C. R. Oz M. (2007). The endocannabinoid anandamide inhibits the function of alpha4beta2 nicotinic acetylcholine receptors. Mol. Pharmacol. 72 (4), 1024–1032. 10.1124/mol.107.036939 17628012

[B149] Storozhuk M. V. Zholos A. V. (2018). TRP channels as novel targets for endogenous ligands: Focus on endocannabinoids and nociceptive signalling. Curr. Neuropharmacol. 16 (2), 137–150. 10.2174/1570159x15666170424120802 28440188 PMC5883376

[B150] Tapper A. R. McKinney S. L. Nashmi R. Schwarz J. Deshpande P. Labarca C. (2004). Nicotine activation of alpha4* receptors: Sufficient for reward, tolerance, and sensitization. Science 306 (5698), 1029–1032. 10.1126/science.1099420 15528443

[B151] Thompson A. J. Lummis S. C. (2007). The 5-HT3 receptor as a therapeutic target. Expert Opin. Ther. Targets 11 (4), 527–540. 10.1517/14728222.11.4.527 17373882 PMC1994432

[B152] Tian X. Pavlopoulos S. Yang D. P. Makriyannis A. (2011). The interaction of cannabinoid receptor agonists, CP55940 and WIN55212-2 with membranes using solid state 2H NMR. Biochim. Biophys. Acta 1808 (9), 2095–2101. 10.1016/j.bbamem.2010.11.026 21129361 PMC3697748

[B153] Tiburu E. K. Bass C. E. Struppe J. O. Lorigan G. A. Avraham S. Avraham H. K. (2007). Structural divergence among cannabinoids influences membrane dynamics: A 2H solid-state NMR analysis. Biochim. Biophys. Acta 1768 (9), 2049–2059. 10.1016/j.bbamem.2007.04.023 17555706

[B154] Turkanis S. A. Karler R. (1986). Effects of delta-9-tetrahydrocannabinol, 11-hydroxy-delta-9-tetrahydrocannabinol and cannabidiol on neuromuscular transmission in the frog. Neuropharmacology 25 (11), 1273–1278. 10.1016/0028-3908(86)90147-4 3025765

[B155] Turner S. E. Williams C. M. Iversen L. Whalley B. J. (2017). Molecular pharmacology of phytocannabinoids. Prog. Chem. Org. Nat. Prod. 103, 61–101. 10.1007/978-3-319-45541-9_3 28120231

[B156] Vijayaraghavan S. Huang B. Blumenthal E. M. Berg D. K. (1995). Arachidonic acid as a possible negative feedback inhibitor of nicotinic acetylcholine receptors on neurons. J. Neurosci. 15 (5), 3679–3687. 10.1523/jneurosci.15-05-03679.1995 7751938 PMC6578235

[B157] Vitale R. M. Iannotti F. A. Amodeo P. (2021). The (Poly)Pharmacology of cannabidiol in neurological and neuropsychiatric disorders: Molecular mechanisms and targets. Int. J. Mol. Sci. 22 (9), 4876. 10.3390/ijms22094876 34062987 PMC8124847

[B158] Wells M. M. Tillman T. S. Mowrey D. D. Sun T. Xu Y. Tang P. (2015). Ensemble-based virtual screening for cannabinoid-like potentiators of the human glycine receptor α1 for the treatment of pain. J. Med. Chem. 58 (7), 2958–2966. 10.1021/jm501873p 25790278 PMC4414066

[B159] Wiley J. L. Martin B. R. (1999). Effects of SR141716A on diazepam substitution for delta9-tetrahydrocannabinol in rat drug discrimination. Pharmacol. Biochem. Behav. 64 (3), 519–522. 10.1016/s0091-3057(99)00130-6 10548265

[B160] Wiley J. L. Marusich J. A. Huffman J. W. (2014). Moving around the molecule: Relationship between chemical structure and *in vivo* activity of synthetic cannabinoids. Life Sci. 97 (1), 55–63. 10.1016/j.lfs.2013.09.011 24071522 PMC3944940

[B161] Wright S. Guy G. (2014). “Licensed cannabis-based medicines: Benefits and risks,” in Handbook of cannabis. Editor Pertwee R. G. (Oxford, UK: Oxford University Press), 373–392.

[B162] Xiong W. Chen S. R. He L. Cheng K. Zhao Y. L. Chen H. (2014). Presynaptic glycine receptors as a potential therapeutic target for hyperekplexia disease. Nat. Neurosci. 17 (2), 232–239. 10.1038/nn.3615 24390226 PMC4019963

[B163] Xiong W. Cheng K. Cui T. Godlewski G. Rice K. C. Xu Y. (2011a). Cannabinoid potentiation of glycine receptors contributes to cannabis-induced analgesia. Nat. Chem. Biol. 7 (5), 296–303. 10.1038/nchembio.552 21460829 PMC3388539

[B164] Xiong W. Cui T. Cheng K. Yang F. Chen S. R. Willenbring D. (2012a). Cannabinoids suppress inflammatory and neuropathic pain by targeting α3 glycine receptors. J. Exp. Med. 209 (6), 1121–1134. 10.1084/jem.20120242 22585736 PMC3371734

[B165] Xiong W. Hosoi M. Koo B. N. Zhang L. (2008). Anandamide inhibition of 5-HT3A receptors varies with receptor density and desensitization. Mol. Pharmacol. 73 (2), 314–322. 10.1124/mol.107.039149 17993512

[B166] Xiong W. Koo B. N. Morton R. Zhang L. (2011b). Psychotropic and nonpsychotropic cannabis derivatives inhibit human 5-HT(3A) receptors through a receptor desensitization-dependent mechanism. Neuroscience 184, 28–37. 10.1016/j.neuroscience.2011.03.066 21477640 PMC3100474

[B167] Xiong W. Wu X. Li F. Cheng K. Rice K. C. Lovinger D. M. (2012b). A common molecular basis for exogenous and endogenous cannabinoid potentiation of glycine receptors. J. Neurosci. 32 (15), 5200–5208. 10.1523/jneurosci.6347-11.2012 22496565 PMC3334839

[B168] Yang D. P. Mavromoustakos T. Beshah K. Makriyannis A. (1992). Amphipathic interactions of cannabinoids with membranes. A comparison between delta 8-THC and its O-methyl analog using differential scanning calorimetry, X-ray diffraction and solid state 2H-NMR. Biochim. Biophys. Acta 1103 (1), 25–36. 10.1016/0005-2736(92)90053-o 1309660

[B169] Yang K. H. Galadari S. Isaev D. Petroianu G. Shippenberg T. S. Oz M. (2010a). The nonpsychoactive cannabinoid cannabidiol inhibits 5-hydroxytryptamine3A receptor-mediated currents in *Xenopus laevis* oocytes. J. Pharmacol. Exp. Ther. 333 (2), 547–554. 10.1124/jpet.109.162594 20160007 PMC2872948

[B170] Yang K. H. Isaev D. Morales M. Petroianu G. Galadari S. Oz M. (2010b). The effect of Δ9-tetrahydrocannabinol on 5-HT3 receptors depends on the current density. Neuroscience 171 (1), 40–49. 10.1016/j.neuroscience.2010.08.044 20800662

[B171] Yang K. Lei G. Xie Y. F. MacDonald J. F. Jackson M. F. (2014). Differential regulation of NMDAR and NMDAR-mediated metaplasticity by anandamide and 2-AG in the hippocampus. Hippocampus 24 (12), 1601–1614. 10.1002/hipo.22339 25087967

[B172] Yang Z. Aubrey K. R. Alroy I. Harvey R. J. Vandenberg R. J. Lynch J. W. (2008). Subunit-specific modulation of glycine receptors by cannabinoids and N-arachidonyl-glycine. Biochem. Pharmacol. 76 (8), 1014–1023. 10.1016/j.bcp.2008.07.037 18755158

[B173] Yano H. Adhikari P. Naing S. Hoffman A. F. Baumann M. H. Lupica C. R. (2020). Positive allosteric modulation of the 5-HT(1A) receptor by indole-based synthetic cannabinoids abused by humans. ACS Chem. Neurosci. 11 (10), 1400–1405. 10.1021/acschemneuro.0c00034 32324370 PMC8275447

[B174] Yao L. Liu C. Wang N. Du F. Fan S. Guo Y. (2020a). Cholesterol regulates cannabinoid analgesia through glycine receptors. Neuropharmacology 177, 108242. 10.1016/j.neuropharm.2020.108242 32712277

[B175] Yao L. Wells M. Wu X. Xu Y. Zhang L. Xiong W. (2020b). Membrane cholesterol dependence of cannabinoid modulation of glycine receptor. Faseb J. 34 (8), 10920–10930. 10.1096/fj.201903093R 32608538 PMC8147113

[B176] Yatsenko N. M. Lozovaya N. A. (2007). Effect of cannabinoids on glycine-activated currents in pyramidal neurons of the rat Hippocampus. Neurophysiology 39 (1), 13–19. 10.1007/s11062-007-0003-z

[B177] Yévenes G. E. Zeilhofer H. U. (2011). Molecular sites for the positive allosteric modulation of glycine receptors by endocannabinoids. PLoS One 6 (8), e23886. 10.1371/journal.pone.0023886 21901142 PMC3162021

[B178] Yu Y. Yang Z. Jin B. Qin X. Zhu X. Sun J. (2020). Cannabidiol inhibits febrile seizure by modulating AMPA receptor kinetics through its interaction with the N-terminal domain of GluA1/GluA2. Pharmacol. Res. 161, 105128. 10.1016/j.phrs.2020.105128 32805354

[B179] Zagzoog A. Mohamed K. A. Kim H. J. J. Kim E. D. Frank C. S. Black T. (2020). *In vitro* and *in vivo* pharmacological activity of minor cannabinoids isolated from Cannabis sativa. Sci. Rep. 10 (1), 20405. 10.1038/s41598-020-77175-y 33230154 PMC7684313

[B180] Zhang L. Oz M. Stewart R. R. Peoples R. W. Weight F. F. (1997). Volatile general anaesthetic actions on recombinant nACh alpha 7, 5-HT3 and chimeric nACh alpha 7-5-HT3 receptors expressed in Xenopus oocytes. Br. J. Pharmacol. 120 (3), 353–355. 10.1038/sj.bjp.0700934 9031735 PMC1564489

[B181] Zhang L. Oz M. Weight F. F. (1995). Potentiation of 5-HT3 receptor-mediated responses by protein kinase C activation. Neuroreport 6 (10), 1464–1468. 10.1097/00001756-199507100-00025 7488749

[B182] Zhu P. J. Lovinger D. M. (2005). Retrograde endocannabinoid signaling in a postsynaptic neuron/synaptic bouton preparation from basolateral amygdala. J. Neurosci. 25 (26), 6199–6207. 10.1523/jneurosci.1148-05.2005 15987949 PMC1352167

[B183] Zimmer A. Zimmer A. M. Hohmann A. G. Herkenham M. Bonner T. I. (1999). Increased mortality, hypoactivity, and hypoalgesia in cannabinoid CB1 receptor knockout mice. Proc. Natl. Acad. Sci. U. S. A. 96 (10), 5780–5785. 10.1073/pnas.96.10.5780 10318961 PMC21937

[B184] Zoli M. Pistillo F. Gotti C. (2015). Diversity of native nicotinic receptor subtypes in mammalian brain. Neuropharmacology 96, 302–311. 10.1016/j.neuropharm.2014.11.003 25460185

[B185] Zou G. Xia J. Han Q. Liu D. Xiong W. (2020a). The synthetic cannabinoid dehydroxylcannabidiol restores the function of a major GABA(A) receptor isoform in a cell model of hyperekplexia. J. Biol. Chem. 295 (1), 138–145. 10.1074/jbc.RA119.011221 31757808 PMC6952599

[B186] Zou G. Zuo X. Chen K. Ge Y. Wang X. Xu G. (2020b). Cannabinoids rescue cocaine-induced seizures by restoring brain Glycine receptor dysfunction. Cell. Rep. 30 (12), 42094209–42094219. 10.1016/j.celrep.2020.02.106 32209479

